# Adjustable Functionalization of Hyper-Cross-Linked Polymers of Intrinsic Microporosity for Enhanced CO_2_ Adsorption and Selectivity over N_2_ and CH_4_

**DOI:** 10.1021/acsami.2c02604

**Published:** 2022-04-26

**Authors:** Haoli Zhou, Christopher Rayer, Ariana R. Antonangelo, Natasha Hawkins, Mariolino Carta

**Affiliations:** † Department of Chemistry, Swansea University, College of Science, Grove Building, Singleton Park, Swansea SA2 8PP, U.K.; ‡ State Key Laboratory of Materials-Oriented Chemical Engineering, College of Chemical Engineering, Nanjing Tech University, 5 Xinmofan Road, Nanjing 210009, P. R. China

**Keywords:** polymers of intrinsic microporosity, isothermal gas adsorption, pore size distribution, selectivity, isosteric heat

## Abstract

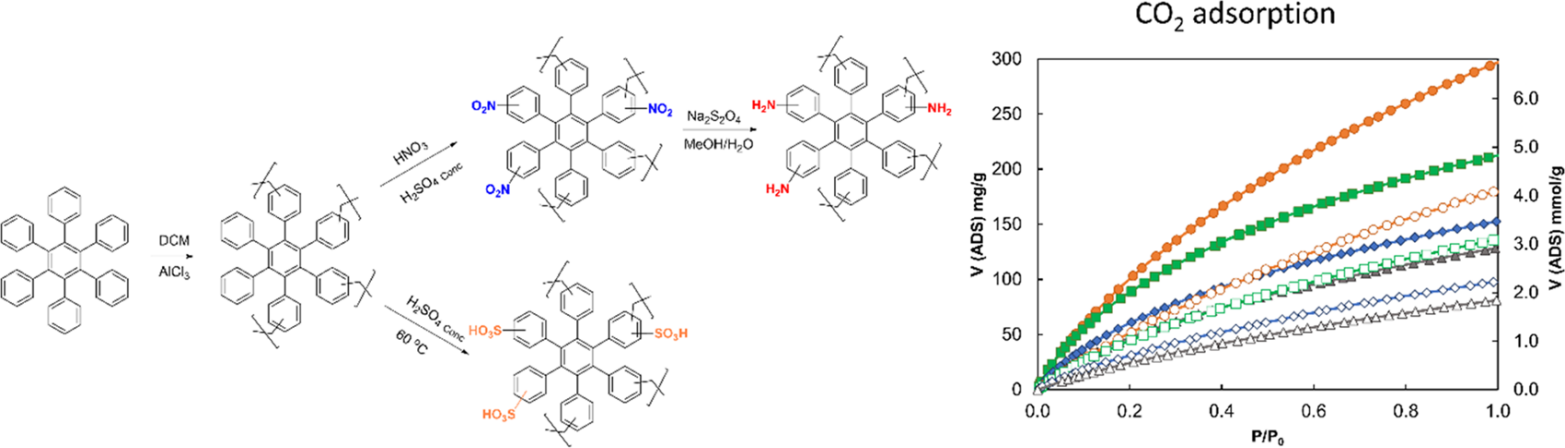

In this paper, we report the design, synthesis, and characterization of a series of hyper-cross-linked polymers of intrinsic microporosity (PIMs), with high CO_2_ uptake and good CO_2_/N_2_ and CO_2_/CH_4_ selectivity, which makes them competitive for carbon capture and biogas upgrading. The starting hydrocarbon polymers’ backbones were functionalized with groups such as −NO_2_, −NH_2_, and −HSO_3_, with the aim of tuning their adsorption selectivity toward CO_2_ over nitrogen and methane. This led to a significant improvement in the performance in the potential separation of these gases. All polymers were characterized via Fourier transform infrared (FTIR) spectroscopy and ^13^C solid-state NMR to confirm their molecular structures and isothermal gas adsorption to assess their porosity, pore size distribution, and selectivity. The insertion of the functional groups resulted in an overall decrease in the porosity of the starting polymers, which was compensated with an improvement in the final CO_2_ uptake and selectivity over the chosen gases. The best uptakes were achieved with the sulfonated polymers, which reached up to 298 mg g^–1^ (6.77 mmol g^–1^), whereas the best CO_2_/N_2_ selectivities were recorded by the aminated polymers, which reached 26.5. Regarding CH_4_, the most interesting selectivities over CO_2_ were also obtained with the aminated PIMs, with values up to 8.6. The reason for the improvements was ascribed to a synergetic contribution of porosity, choice of the functional group, and optimal isosteric heat of adsorption of the materials.

## Introduction

Carbon dioxide is accepted as being the most dominant contributor to global warming.^1^ According to a recent report from NASA,^2^ the concentration of CO_2_ in the atmosphere recorded to date (last data from 2021) is greater than at any other time in modern history, exceeding 417 ppm and rising. This roughly translates into a ∼49% increase since the beginning of the industrial age (280 ppm in ∼1850) and a 13% increase since 2000, when it was already close to 370 ppm. It is widely recognized that we are at its highest point in over 20,000 years, as it is calculated that the concentration of CO_2_ at the end of the last glacial age was ∼185 ppm. There is also an overwhelming scientific consensus (18 of the best recognized scientific associations) that the steep increase in the concentration is largely attributable to human activities.^3^

Sensibly, governments, industries, and academia started addressing the problem a long time ago, implementing new policies aimed at reducing the release of this greenhouse gas into the environment via new carbon capture and sequestration schemes (CCS).^4^ Various systems have been developed to either retrofit existing chemical plants, with modules intended to trap CO_2_ before it is released into the atmosphere (postcombustion carbon capture, the most common) or by removing it once already released via direct air carbon capture (DACCS).^5,6^ The latter is a fascinating option but it is also very difficult to be accomplished, as the concentration of CO_2_ in the air is extremely low for current techniques (just over 400 ppm).^7^ The former is certainly more feasible for the industry, for instance, by loading a variety of adsorbents in pre-existing plants and capturing CO_2_ via temperature (TSA),^8^ pressure (PSA),^9^ or vacuum swing (VSA)^10^ adsorption. The overall operating costs of these techniques are regarded as cheaper than the typical amine-bed carbon capture.^11^ In fact, to date, the removal of CO_2_ from flue gas is mainly achieved by its absorption onto aqueous amine solutions, mainly due to their affinity for slightly Lewis-acid gases like CO_2_. However, the energy penalty of their utilization is strongly affected by the high isosteric heat of adsorption (*Q*_st_) of CO_2_, which means that its desorption after capture is very energy-demanding, and that makes the amine beds difficult to be regenerated.^12^ Researchers suggest *Q*_st_ values of about 50 kJ mol^–1^ to define the boundary between chemisorption, where a chemical bond with the gas and the sorbent is achieved, and physisorption, where the gas is simply “mechanically” trapped within pores (the energy of physisorption is comparable to van der Waals (vdW) forces).^13^ This definition is not conclusive, anyway, as several parameters need to be considered at the same time to have a full picture.^14^ The general conclusion is that, if the gas can diffuse rapidly within the pores, a high *Q*_st_ guarantees a quick CO_2_ capture but it also requires more energy for its desorption, whereas a low *Q*_st_ grants a quick release but more adsorption cycles are necessary to achieve an acceptable removal performance. It is evident that a good compromise between the two will produce an ideal material for carbon capture. This means that low/medium *Q*_st_ (between 20 and 35 kJ mol^–1^, so still in the physisorption range) are desirable for carbon capture, preferentially via PSA or VSA.^15^ This could be accomplished by producing highly porous materials (so, promoting physisorption) and decorating them with functional groups that enhance their affinity for CO_2_ (i.e., keeping the chemisorption to acceptable values). As a good example of such a compromise, trapping CO_2_ using ionic liquids (ILs) represents an emerging CCS technique. ILs are often embedded in porous materials to combine the advantages provided by their low vapor pressure, tunable structures, and the presence of a permanent charge that increases their capability to trap CO_2_ efficiently. In addition, the porosity of the support maximizes the contact of the gas within their structures.^16^ For this reason, it is not uncommon to see IL composite systems for CO_2_ capture blended with metal–organic frameworks (MOFs)^17^ or other porous polymeric systems.^18−20^ Porous materials such as MOFs seem, in fact, very attractive for CCS^21^ because of their high surface areas and the tunability of their structures that allows the introduction of functional groups. However, the coordinated metals enclosed in their backbones make them less attractive from the environmental point of view. Insoluble amorphous materials, such as hyper-cross-linked polymers, are a valid alternative to MOFs, especially considering their metal-free structures. To be competitive for carbon capture, the working capacity of these sorbents should result in greater than 2 mmol g^–1^ (>88 mg g^–1^) at 298 K, they should be very stable and resistant for numerous cycles and, of course, inexpensive and producible on large scale.^15^ Several porous hyper-cross-linked polymers have been investigated for this purpose, and the best performances were achieved when functional groups such as hydroxy (−OH),^22,23^ amino (−NH_2_),^24−26^ nitro (−NO_2_),^27^ and sulfonic (−SO_3_H)^28,29^ were incorporated in the backbone, which improved the affinity of the material for CO_2_.

Polymers of intrinsic microporosity (PIMs) represent an emerging family of porous polymers. They are materials in which porosity originates from the appropriate choice of the monomers, which leads to an inefficient packing of the polymer chains in the solid state and to the formation of voids of nanodimension.^30^ They have proved to be excellent in separating CO_2_ from other gases due to a combination of a narrow pore size, which leads to excellent molecular sieving properties, and high solubility of CO_2_ in their backbones.^31−33^

In fact, they typically exhibit pore sizes between 3.5 and 8.5 Å^34^ so that CO_2_, which has a kinetic diameter of 3.3 Å, can interact with both sidewalls of the pores.^35^ However, the main advantage of PIMs probably lies in the ease of their synthesis and functionalization, which consents to introduce polar groups that improve the affinity for CO_2_.^36^ Recently, the synthesis of a series of new hyper-cross-linked PIMs with very high Brunauer–Emmett–Teller (BET) surface areas (SA_BET_ up to 2435 m^2^ g^–1^) was reported by Msayib and McKeown.^37^ They are prepared from hydrocarbon monomers and polymerized via a simple and efficient Friedel–Crafts reaction.

The great potential of these knitted polymers and the simplicity of the reaction was further demonstrated by Lau et al., who reported the synthesis of a triptycene hyper-cross-linked PIM via flow chemistry, also proving the possible scalability of the method.^38^ In a recent work, we reported the functionalization of two of these PIMs with sulfonic groups, which proved very effective in selectively removing antidepressants from wastewater.^39^

In this paper, we used the same approach systematically synthesizing and functionalizing a series of hydrocarbon-based PIMs, namely hexaphenylbenzene (**HPB**), triphenylbenzene (**TPB**), spirobisfluorene (**SBF**), and triptycene (**Trip**), with amino (−NH_2_), nitro (−NO_2_), and sulfonic groups (−SO_3_H), focusing the study on the assessment of their CO_2_ uptake and their selectivity against N_2_ and CH_4_ to elucidate their potential for CCS and biogas upgrading.

## Results and Discussion

### Synthesis and Functionalization of Polymers

The synthesis of the hydrocarbon hyper-cross-linked polymers was performed following the procedure reported by Msayib and McKeown.^37^ The reaction consists of a Friedel–Crafts polymerization of the monomers, catalyzed by AlCl_3_ in the presence of dichloromethane (DCM), which acts as both the solvent and as a cross-linker bidentate ligand.

**Figure 1:**
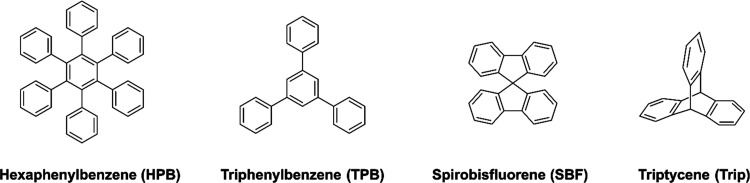
Monomers used in this work to prepare the hydrocarbon-based hyper-cross-linked PIMs.

**Figure 2:**
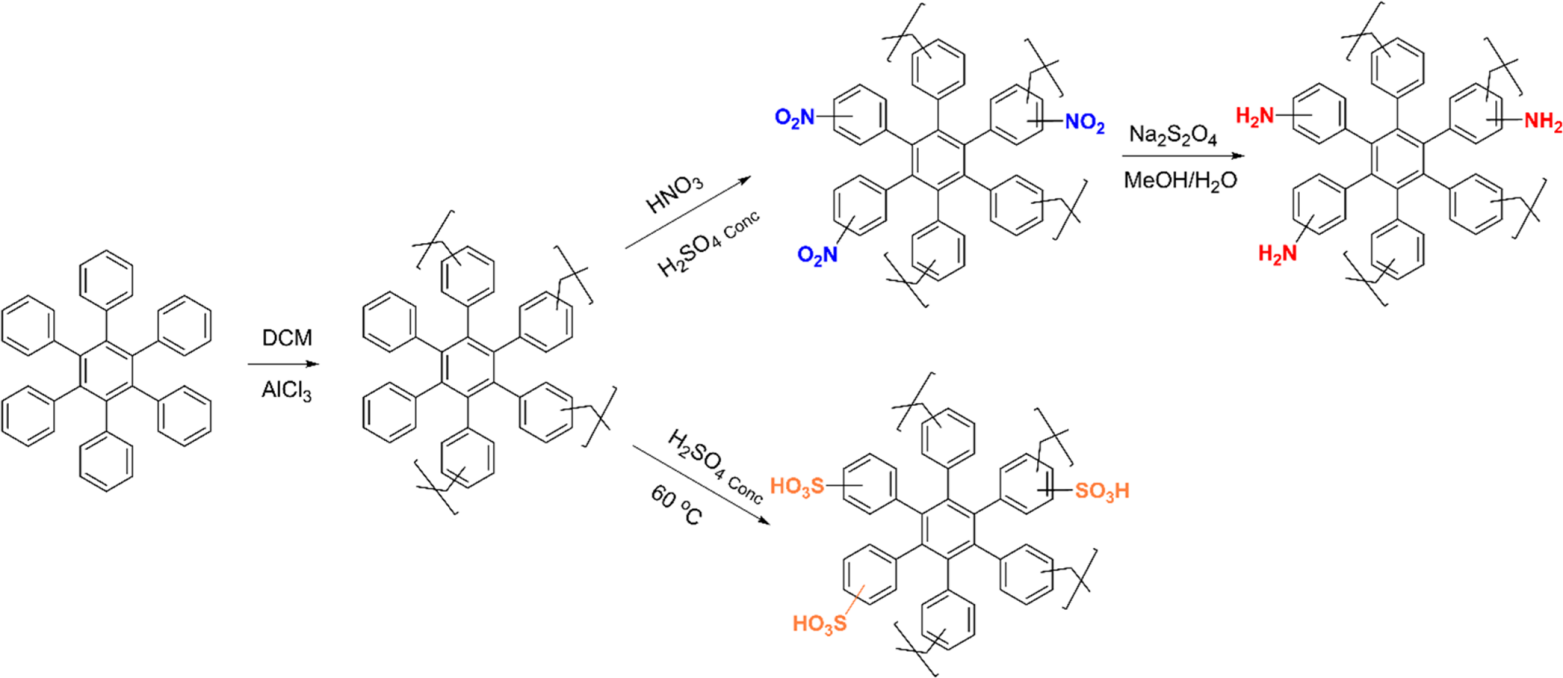
Synthesis and postpolymerization modification of **HPB-PIM**.

We chose to use only monomers known to induce high BET surface areas (SA_BET_) in the final material (Figure 1). Starting from a high microporosity is crucial, especially considering that the introduction of functional groups in the preformed backbone may cause the loss of some of it due to a known “pore filling” effect.^40,41^ So, if we want to retain some of the original internal free volume (IFV), which is deemed necessary to improve the gas separation performance by molecular sieving, it is essential to start from materials with a very high surface area. As anticipated, despite the loss of porosity, we trusted that the addition of polar groups would enhance the affinity of the material for CO_2_, with an improvement in the selectivity toward larger gases such as N_2_ and CH_4_.^42,43^ The fastest way to introduce such groups is by inserting them via postpolymerization modifications of their aromatic structures. For instance, we thought to incorporate either the basic amines (via nitration, followed by their reduction)^44^ or acidic groups via direct sulfonation.^39^ In a recent paper, we reported the successful addition of sulfonic groups to two hyper-cross-linked hydrocarbon polymers (**TPB** and **TRIP**) by simply reacting the preformed backbone with concentrated sulfuric acid at 60 °C and obtaining an average of three acid groups per repeated units, as proven by acid/base titration.^39,45^ The same procedure was herein used to also sulfonate the **SBF** and **HPB** polymers. The introduction of the basic functionalities was achieved by nitration of the aromatic moieties with HNO_3_ catalyzed by H_2_SO_4_, followed by the reduction of the nitro groups with Na_2_S_2_O_4_ in a mixture of alcohol/water. Figure 2 shows the scheme of a typical procedure, in this case, using **HPB** as an example of the hydrocarbon backbone. All of the reported polymers produced similar results in terms of both yields and purity, which proves the validity and reproducibility of our protocols. Details about the synthesis for all of the polymers are reported in the Supporting Information (SI).

### Structural Characterization of the Functionalized Polymers

**Figure 3:**
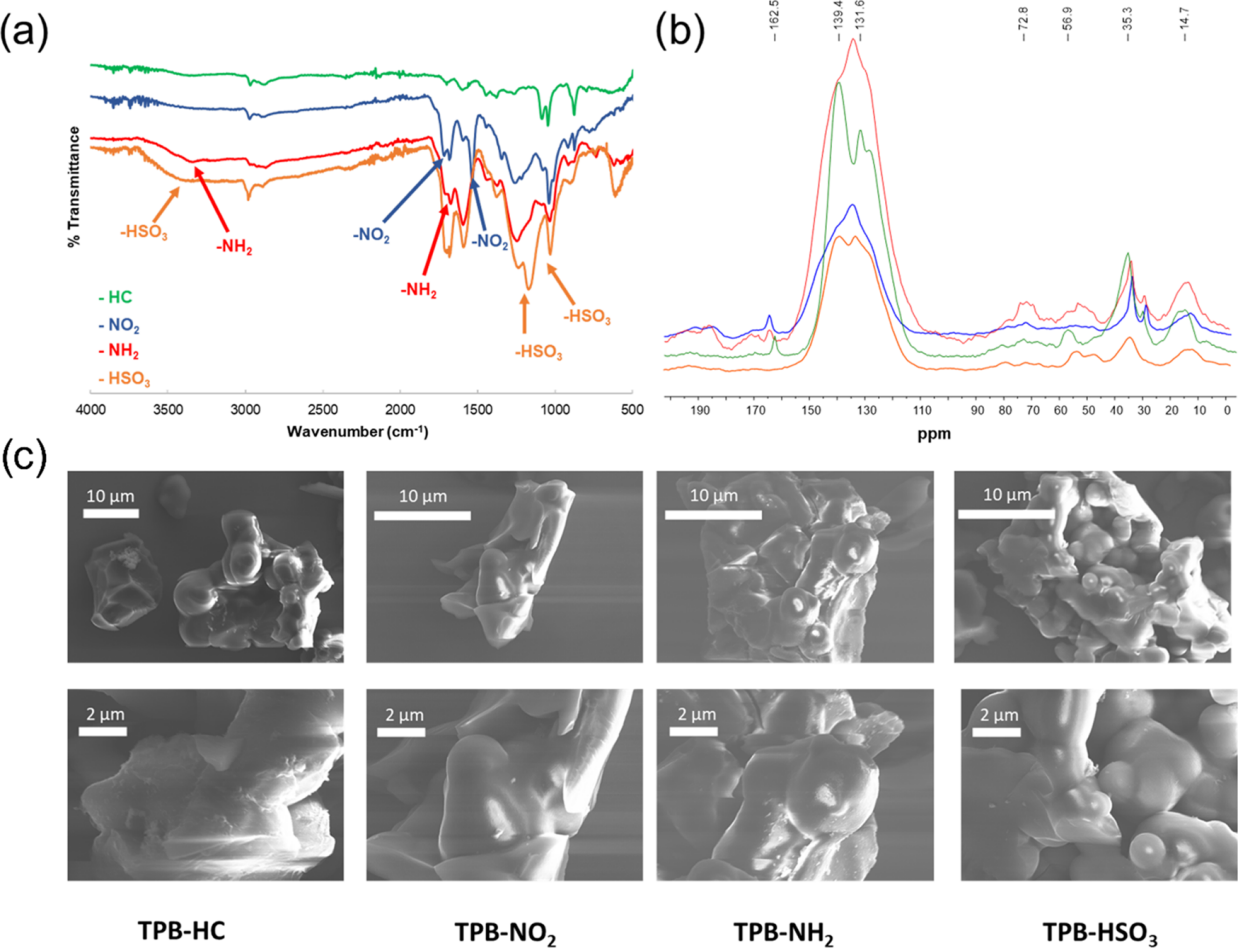
(a) FTIR, (b) ^13^C SSNMR, and (c) SEM images of **TPB-PIMs**.

As expected from the type of polymerization, all of the synthesized hyper-cross-linked PIMs were found completely insoluble in common organic solvents, which prevented their characterization via the typical solution-based techniques, i.e.,^1^H NMR, needed for confirmation of the composition of the backbone, or gel permeation chromatography (GPC), typically used for the estimation of the molecular mass of the chains. The carbon contents could be, in theory, determined by elemental analysis but they were always proved lower than the expected one. This is something that is frequently observed in highly porous aromatic polymers where water molecules and other adsorbed gas may change the ratios, which makes this technique poorly reproducible.^46−48^ The correct composition was demonstrated by Fourier transform infrared (FTIR) spectroscopy and solid-state ^13^C NMR (SSNMR), which proved the efficient incorporation of the different functional groups. As an example, Figure 3a shows the overlay of the FTIR spectra of the **TPB-PIMs**. The bands around 3300 and 1550 cm^–1^ confirm the presence of the −NH_2_ groups on the aminated materials, whereas peaks centered at ∼3450, ∼1200, and ∼1000 cm^–1^ show the typical signatures of the −HSO_3_ groups of the sulfonated version. The FTIR of the nitrated TPB polymer is very similar to the aminated version, apart from the obvious lack of −NH_2_ peaks. Figure 3b shows the overlay of the ^13^C SSNMR of the same TPB polymers, which was used to further validate the correct structure of the hyper-cross-linked PIMs.

As expected, the broad peak in the area around 160 and 130 ppm confirms the presence of aromatic carbons, and several smaller peaks, in the range between 75 and 15 ppm, suggest the presence of the methylene cross-linkers, some of which likely shifted due to the vicinity of a polar functional group. FTIR and ^13^C SSNMR spectra for all of the other single polymers are shown in the SI (Figure SI12–15 for FTIR and Figure SI16–31 for ^13^C SSNMR). Scanning electron microscopy (SEM) images (Figure 3c) were taken to investigate potential changes in the morphology of the material due to the insertion of different functionalities. The analysis of the images at different magnifications supports the formation of amorphous materials very similar to one another. In fact, apart from **TPB-HC**, which seems to exhibit a slightly larger particle size than the other three, all polymers seem to display globular particles of around 1–2 μm in size. All of the other functionalized polymers herein reported followed the same trend indicated by **TPB-PIMs**, which is also in line with similar published materials,^29,49,50^ proving the reproducibility of our methods.

### Physical Characterization

**Figure 4:**
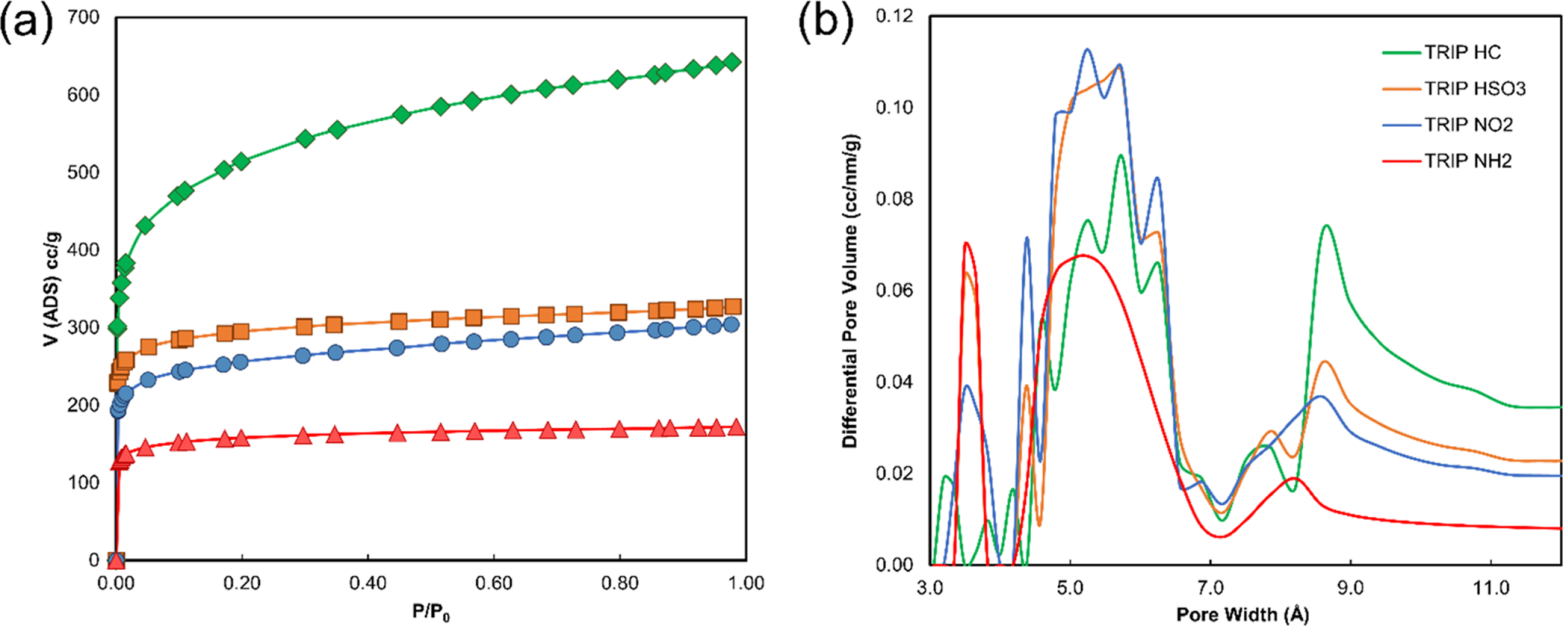
(a) Overlay of N_2_ adsorption isotherms (77 K) and (b) NLDFT pore size distribution calculation (from CO_2_ adsorption at 273 K) for **PIM-TRIP** polymers. Desorption curves were removed for clarity.

The measurement of the microporosity was performed via the typical isothermal N_2_ adsorption at 77 K, with the calculation of the individual apparent BET surface areas (SA_BET_). All of the hydrocarbon-based PIMs proved to be highly microporous, with a type I(a) isotherm that denotes a steep uptake at low partial pressure, typical of ultramicroporous polymers. The lack of a pronounced hysteresis (Figures SI3, SI7, SI5, and SI9) hints at small and interconnected pores similar to microporous carbons.^51^ The SA_BET_ ranges from **TPB** (2540 m^2^ g^–1^) > **HPB** (1933 m^2^ g^–1^) > **TRIP** (1880 m^2^ g^–1^) > **SBF** (1604 m^2^ g^–1^), which is similar to the ones previously reported by Msayib and McKeown.^37^Figure 4a shows the overlay of the N_2_ adsorption isotherms for the **PIM-TRIP** family.

The pore size distribution analysis, calculated via nonlocal density functional theory (NLDFT) from the CO_2_ adsorption at 273 K, provided values in the typical range of PIMs. The first set of pores centered around 3.5 Å confirmed the ultramicroporosity of these materials, followed by two more peaks centered around 5.6 and 8.6 Å. Despite the fact that pore size distribution (PSD) assessed via gas adsorption is known to be mostly qualitative,^52^ it is worth noting that the contribution coming from the larger peak (8.6 Å) is more evident for the pure hydrocarbon polymers and decreases for the functionalized ones (**TRIP-PIMs** in Figure 4b). This suggests that the substitution leads to a general shrinking of the pores that move, predominantly, toward the ultramicroporous region. This feature is very important for the understanding of the separation of CO_2_ from N_2_ and CH_4_, as it will not be dominated only by the enhanced affinity due to the introduction of substituents, but also by an improved molecular sieving effect. The physical data for all polymers are shown in Table 1, whereas the single isotherms and the pore size distribution (PSD) are reported in the SI (Figure SI3–10). The postpolymerization modifications led to the expected loss of porosity, but not all of the functionalities had the same impact. In fact, both nitro- and sulfonic-containing polymers retained a good amount of the initial porosity, with **PIM-NO**_**2**_ ranging from 909 to 1286 m^2^ g^–1^ and **PIM-HSO**_**3**_ from 1145 to 1390 m^2^ g^–1^. This suggests that the introduction of such groups leads to the loss of some of the internal free volume but, apart from the expected pore-filling effect, there are no other contributing factors that reduce the overall porosity. **PIM-NH**_**2**_, on the other hand, provided lower SA_BET_ values, ranging from 610 to 997 m^2^ g^–1^, showing a more pronounced loss of porosity compared to the other two families of substituted PIMs. This behavior is not uncommon for aminated porous polymers^24,44,53^ and suggests that the presence of amines affects the overall porosity more than other groups, most likely promoting stronger intermolecular hydrogen bonding that pulls the polymer chains closer together.^54^

**Table 1:** Physical Characterization of Polymers and Gas Selectivity

			CO_2_ adsorption		IAST selectivity^b^		
Polymer	BET (m^2^ g^–1^)	Pore volume^a^ (cc g^–1^)	273 K (1 bar) (mg g^–1^) (mmol g^–1^)	298 K (1 bar) (mg g^–1^) (mmol g^–1^)	CO_2_/N_2_	CH_4_/CO_2_	*Q*_st_^c^ (KJ mol^–1^)
This Work							
Hydrocarbon							
PIM-HPB	1933	1.63	137 (3.12)	69 (1.57)	13.5		21.6
PIM-SBF	1604	0.837	144 (3.28)	79 (1.79)	11.4		23.3
PIM-TPB	2540	1.300	220 (5.00)	121 (2.75)	14.1	3.1	25.2
PIM-Tript	1880	0.996	166 (3.78)	109 (1.48)	13.9	3.6	24.2
Nitrated							
PIM-HPB-NO_2_	1286	1.24	137 (3.11)	88 (2.00)	26.5	7.1	29.5
PIM-SBF-NO_2_	909	0.57	147 (3.34)	98 (2.23)	23.4	6.8	30.1
PIM-TPB-NO_2_	950	0.553	225 (5.13)	137 (3.11)	24.7	8.0	32.1
PIM-Tript- NO_2_	975	0.472	214 (4.87)	115 (2.61)	24.8	6.8	34.5
Aminated							
PIM-HPB-NH_2_	997	0.969	123 (2.80)	81 (1.84)	21.6	7.0	30.4
PIM-SBF-NH_2_	669	0.303	128 (2.90)	97 (2.20)	24.2	6.3	27.7
PIM-TPB-NH_2_	710	0.333	196 (4.45)	131 (2.98)	26.1	8.6	31.7
PIM-Tript-NH_2_	610	0.270	157 (3.57)	124 (2.81)	25.5	7.4	34.7
Sulfonated							
PIM-HPB-HSO_3_	1390	1.31	128 (2.9)	81 (1.84)	18.7	7.1	27.9
PIM-SBF-HSO_3_	1063	0.557	152 (3.45)	98 (2.23)	23.4	6.4	28.7
PIM-TPB-HSO_3_	1585	0.852	298 (6.77)	179 (4.07)	17.9	7.8	29.0
PIM-Tript-HSO_3_	1145	0.507	216 (4.91)	135 (3.07)	19.2	7.8	30.9
Comparison with Other Polymers							
Ad-MALP-1^25^	1629	0.396	182	89	28.4	5.37	26.7
Ad-MALP-4^25^	1541	0.384	166	78	25.4	4.21	27.4
PI-ADNT^27^	774	0.415	150	85	25	9	35
PI-NO2-1^27^	286	0.155	177	89	18	11	43
TPPA–DMB^64^	883	0.53	124	76	25	5.3	
TATHCP^65^	997	0.63	125	77	22	4.8	33
NPC-700-KOH^66^	2616	1.14	240	127	21.5		24
HCP2a-K700^67^	1964	1.04	251	134	10.8		24.8
PBZC-3-900^68^	2423	1.47	359	204	31	6.2	35
Polymer 3^69^	1717	0.37	188	103	19.4	4.1	26.5
NPOF-1-NO2^44^	1295	0.36	160	111	20	6	29.2
NPOF-1-NH2^44^	1535	0.48	250	166	25	10	32.1
HCP-SC-SO3H	1246	0.94		62	19		35
C1M3-Al^70^	1783	1.29	181		23.4		20.1

a At *P*/*P*_0_ ∼ 0.98.

b Calculated according to IAST at 298 K and 1 bar.^59,60^

c Isosteric heat of adsorption (in kJ mol^–1^) of corresponding gas at zero coverage calculated from isotherms collected at 273 and 298 K and fitted with the Langmuir–Freundlich equation and calculated via the Clausius Clapeyron equation.

### CO_2_ Adsorption

**Figure 5:**
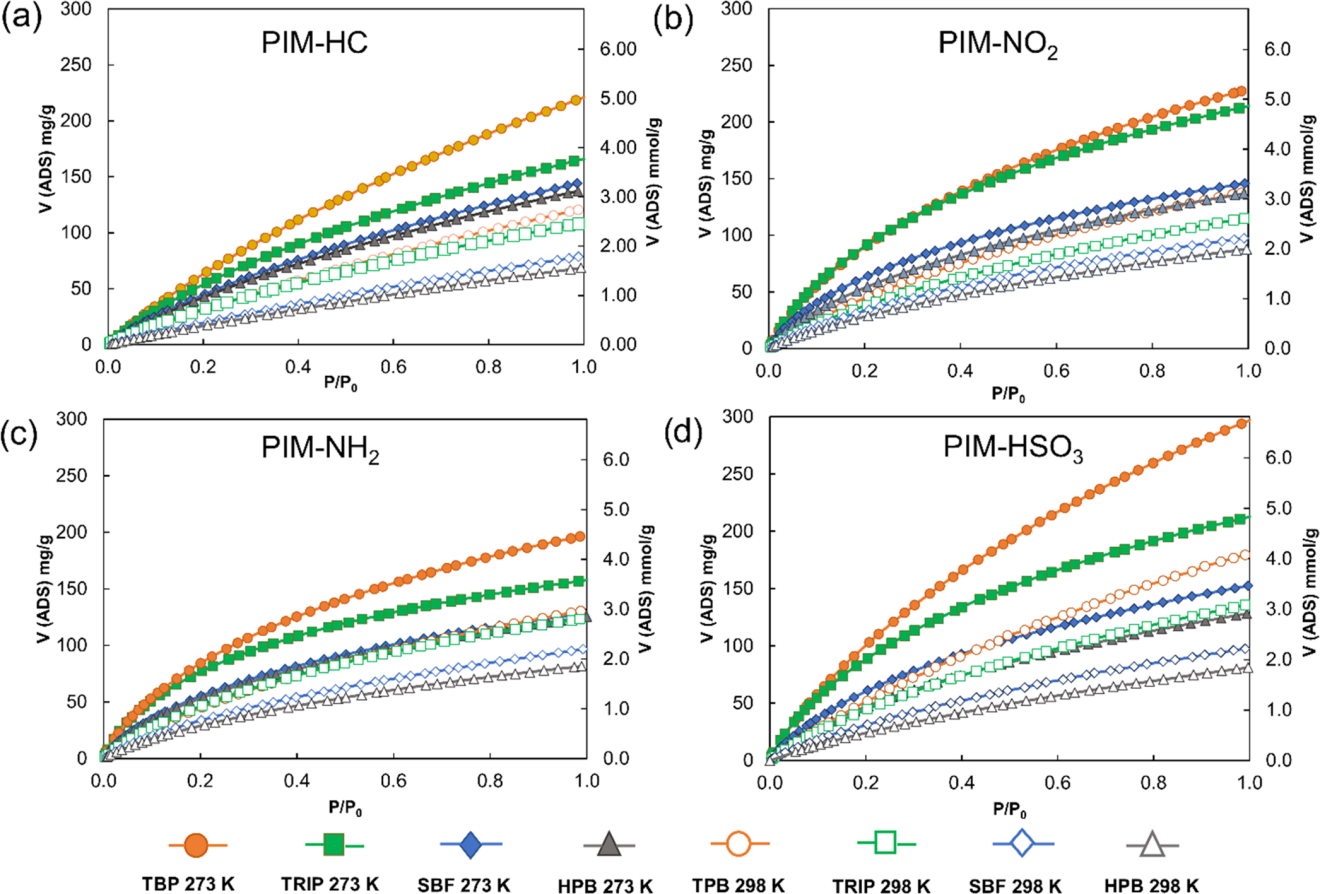
CO_2_ adsorption of hyper-cross-linked PIMs at 273 and 298 K.

The CO_2_ uptake was strongly influenced by the incorporation of functional groups, which altered uptakes and selectivity in the way we expected. From Table 1 and Figure 5, we can see that the CO_2_ uptake at 273 K for all of the hydrocarbon-based PIMs is very high, as the best polymer (**PIM-TPB**) adsorbed 220 mg g^–1^ (5.00 mmol g^–1^) of CO_2_ at 1 bar, followed by **PIM-TRIP** > **PIM-SBF** > **PIM-HPB**. The excellent adsorption was attributed to the very high porosity of the PIMs. Despite producing lower surface areas, the functionalization generated high CO_2_ uptakes for the nitro- and amino-containing PIMs, demonstrating the benefits of having such groups in the backbone, which showed a further improvement after the sulfonation process. **PIM-TPB-HSO**_**3**_, in fact, proved the best of the entire set with a remarkable CO_2_ uptake of 298 mg g^–1^ (6.77 mmol g^–1^) at 273 K and 179 mg g^–1^ (4.06 mmol g^–1^) at 298 K, which makes it competitive with some of the state-of-the-art polymers reported, for comparison, at the bottom of Table 1. All of the reported PIMs showed the same trends, with the sulfonated polymers providing better uptakes than the nitrated, which proved to be slightly better than the aminated versions. The latter came as a surprise as more basic moieties were thought to have a better affinity for CO_2_, which is a weak Lewis acid, but the reduced porosity probably compensated for the higher affinity, reducing the overall performance. The reproducibility of the work clearly shows that the sulfonated and nitrated PIMs are superior, at least in the absence of water/moisture in the adsorbed gas.

### Selectivity Studies

The selectivity of CO_2_ over other gases is undoubtedly as important as the final uptakes, especially when the materials are designed to separate potential mixtures. It is known that removing CO_2_ from any gases is crucial to assessing the performance of the adsorbent materials for carbon capture and sequestration (CCS). The removal of CO_2_ from methane, instead, is an important industrial process, aimed to improve the performance of natural gases when used as fuels (also known as biogas upgrading).^55,56^

**Figure 6:**
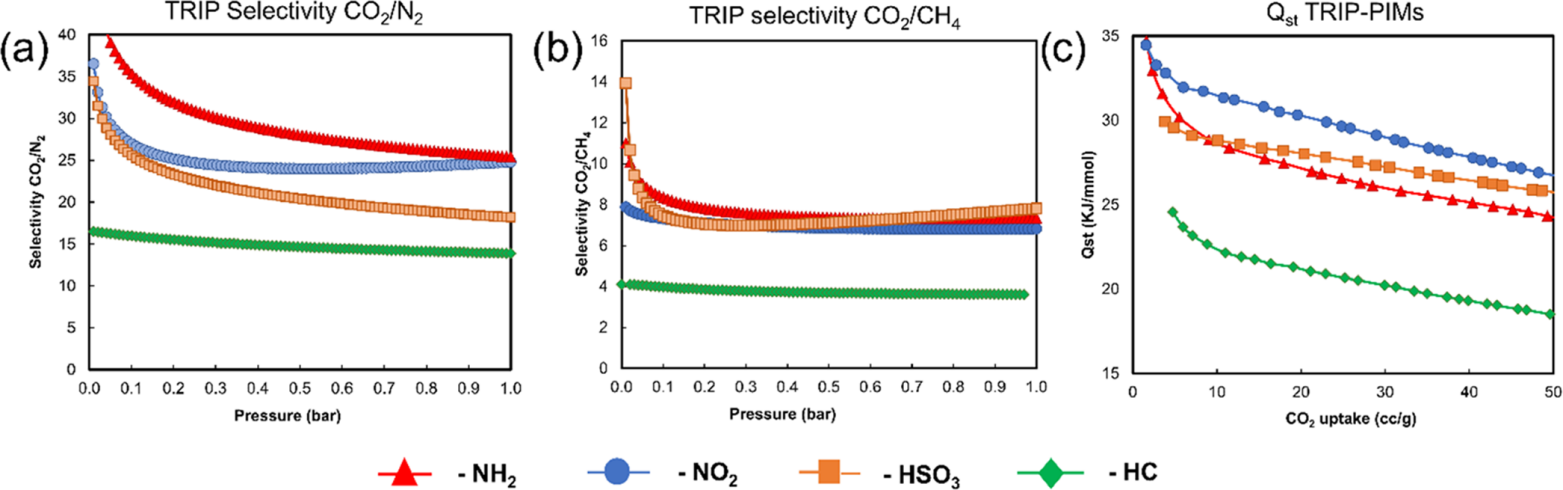
(a) IAST CO_2_/N_2_ selectivity (simulating a 15/85 composition), (b) IAST CO_2_/CH_4_ selectivity (simulating 50/50 composition), and (c) *Q*_st_ of TRIP-PIMs.

All of the studies were conducted by measuring the adsorption of single gases at 298 K and taking the selectivity at 1 bar, so mimicking vacuum swing adsorption conditions (VSA).^57,58^ The potential mixture of the two gases was set at 15/85 for CO_2_/N_2_ or 50/50 CO_2_/CH_4_, using the IAST method to assess the potential separation^59,60^ and simulating the VSA conditions for postcombustion carbon capture and the biogas upgrading. All hydrocarbon polymers proved to be poorly selective toward both N_2_ and CH_4_. This is not unexpected, as the hydrocarbon backbone is rather inert, and we saw that the pores seem slightly larger compared with the substituted versions. As shown in Table 1, despite the relatively lower uptakes, both nitrated and aminated polymers showed better selectivities than sulfonated ones, with CO_2_/N_2_ values ranging from 23.4 to 26.5 (Figure 6a). The CO_2_/CH_4_ selectivity values are typically lower than the corresponding CO_2_/N_2_ for these kinds of porous polymers,^61,62^ yet the results obtained with our hyper-cross-linked PIMs proved competitive with state-of-the-art porous materials also for this separation, as shown in Table 1 and in Figure 6b. Even in this case, the best results were achieved with the aminated PIMs, with **PIM-TPB-NH**_**2**_ that reached a CO_2_/CH_4_ selectivity of 8.6. The nitrated polymers proved to be almost as good as the aminated ones and so did the sulfonated ones. The enhanced selectivity of the substituted polymers could be also explained by analyzing their isosteric heats of adsorption, especially for the aminated and nitrated PIMs (*Q*_st_, in Table 1). In fact, the *Q*_st_ values obtained for these polymers show that their adsorption mechanism fits in between physisorption and chemisorption, although we believe that the values reflect that physisorption is the dominant mechanism, as the highest value (34.7 KJ mol^–1^ for **PIM-Trip-NH**_**2**_) approaches 50 KJ mol^–1^ that is considered as the threshold between the two mechanisms.^12^

As discussed earlier, having a good compromise between physisorption and chemisorption is ideal, as a reasonable *Q*_st_ guarantees good affinity for CO_2_, which improves its separation from other gases. At the same time, staying below 50 KJ mol^–1^ ensures that the energy penalty of its desorption would not be very high.^12,63^Figure 6c shows an example of the selectivity and the *Q*_st_ plots for the **TRIP-PIM** family; the single plots for all of the other polymers are reported in Figure SI11.

## Conclusions

The synthesis and characterization of a series of hyper-cross-linked PIMs prepared via a knitting method promoted by Friedel–Crafts polymerization provided interesting materials for CO_2_ adsorption and selectivity toward N_2_ and CH_4_. The hydrocarbon-based polymers were functionalized postpolymerization to introduce polar groups, which helped us to tune the porosity and the polarity of the backbone, which produced an improvement of the CO_2_ uptakes and the CO_2_/N_2_ and CO_2_/CH_4_ selectivity on the final materials. The data showed that the sulfonation of the hydrocarbon PIMs provided the best results in terms of CO_2_ uptakes and CO_2_/CH_4_ selectivity, whereas the nitrated and aminated PIMs showed enhanced CO_2_/N_2_ selectivity. The new polymers proved to be competitive with state-of-the-art materials, and we believe that this is due to a combination of pore size, functionalization, and isosteric heats of adsorption, which makes them very attractive materials for carbon capture and biogas upgrading applications. The ease of functionalization demonstrates that we can “play” with chemistry to tune the selectivity of these materials for the chosen gases. Finally, the isosteric heats of adsorption analysis suggests an adsorption mechanism in between physisorption and chemisorption, which is ideal for VSA as it assures a good affinity for CO_2_ and a reduced energy penalty of its release.

## Supporting Information Available

Synthesis and characterization of polymers, selectivity CO_2_/N_2_ and CO_2_/CH_4_, isotherms, heats of adsorption, FTIR spectra, and solid-state ^13^C NMR spectra (PDF)
