# Supplementary material for: Adjustable Functionalization of Hyper-Cross-Linked Polymers of Intrinsic Microporosity for Enhanced CO2 Adsorption and Selectivity over N2 and CH4
Source: ACS Appl Mater Interfaces. 2022 Apr 26;14(18):20997–1006. doi: 10.1021/acsami.2c02604 (PMC9100501; doi:10.1021/acsami.2c02604)
Supplement: Supplementary file 1 — am2c02604_si_001.pdf [file am2c02604_si_001.pdf]

Supporting Information

Adjustable Functionalisation of Hypercrosslinked Polymers of Intrinsic Microporosity for Enhanced CO_2 Adsorption and Selectivity over N_2 and CH_4

Haoli Zhou, [a, b] Christopher Rayer, [a] Ariana R. Antonangelo, [a] Natasha Hawkins [a] and Mariolino Carta [a]*

[a] Department of Chemistry, Swansea University, College of Science, Grove Building, Singleton Park, Swansea, SA2 8PP, UK.

[b] State Key Laboratory of Materials-Oriented Chemical Engineering, College of Chemical Engineering, Nanjing Tech University, 5 Xinmofan Road, Nanjing 210009, PR China

Corresponding author's E–mail: mariolino.carta@swansea.ac.uk

Table of content

1. **General methods and equipment** 2
2. **IAST Selectivity calculation.** 3
3. **Experimental part** 3
4. **Selectivity CO_2/N_2** 8
5. **Selectivity CO_2/CH_4** 9
6. **Isotherms** 10
7. **Heats of adsorption** 18
8. **FT-IRs** 19
9. **Solid state ^{13}C NMRs** 21
10. **TGA** 29
11. **References** 31

1. General methods and equipment

Commercially available reagents and gases were used without further purification. All reactions using air/moisture sensitive reagents were performed in oven-dried or flame-dried apparatus, under a nitrogen atmosphere. Low-temperature N_2 (77 K and 298 K), CO_2 (273 K and 298 K) and CH_4 (298 K) adsorption/desorption measurements of PIM powders were made using a Quantachrome Nova-e. Samples were degassed for 800 min at 100 °C under high vacuum prior to analysis. The gases were supplied by BOC and used without any further purification (N_2 purity > 99.999, CO_2 purity > 99.995%). The specimen was measured twice after outgas in two different cells to minimize the error, providing the same results. The data were analysed with the software provided with the instrument. The BET was calculated at a relative pressure $P/P_0 < 0.1$. NLDFT and H-K analysis were performed to calculate the pore size distribution and volume, considering a carbon equilibrium transition kernel at 273 K based on a slit-pore model; the kernel is based on a common, one centre, Lennard-Jones model. To assess the potential chemisorption, heats of adsorption were calculated from the CO_2 curves measured at 237K and 298K. The data were analysed with the QuadraWin software and fitted with the Langmuir-Freundlich equation and calculated via the Clausius-Clapeyron equation. TGAs were performed using the device Thermal Analysis SDT Q600 at a heating rate of 10 °C/min from 30 to 1000 °C. Solid-state ^{13}C NMR spectra were recorded using a Bruker Avance III spectrometer equipped with a wide-bore 9.4 T magnet (Larmor frequencies of 100.9 MHz for ^{13}C). Samples were packed into standard zirconia rotors with 4 mm outer diameter and rotated at a magic angle spinning (MAS) rate of 12.5 kHz. Spectra were recorded with cross polarisation (CP) from 1H using a contact pulse (ramped for 1H) of 1.5 ms. High-power ($\nu_1 \approx$ 100 kHz) TPPM-15 decoupling of 1H was applied during acquisition to improve resolution. Signal averaging was carried out for 6144 transients with a recycle interval

of 2 s. Chemical shifts are reported in ppm relative to $(CH_3)_4Si$ (TMS) using the CH_3 signal of L-alanine (δ = 20.5 ppm) as a secondary solid reference.

2. IAST Selectivity calculation.

The ideal adsorption solution theory (IAST) of Myers and Prausnitz[1] is typically used to the selectivity of binary mixtures of gases from the single isotherms. The isotherms were fitted with Dual-Site Langmuir-Freundlich using the software IAST++ [2] and the selectivity (S) was calculated according to the formula:

$$S = \frac{Q_{CO2}}{Q_{N2}} \times \frac{P_{N2}}{P_{CO2}}$$

Were

- P_{CO2} is the partial pressure of CO_2
- P_{N2} is the partial pressure of N_2
- Q_{N2} is the N_2 uptake
- Q_{CO2} is theCO_2 uptake

3. Experimental part

PIM-TPB, PIM-Tript, PIM-TPB-HSO$_3$ and **PIM-Tript-HSO$_3$** were synthesised according to our previous paper.[3]

PIM-SBF[4]

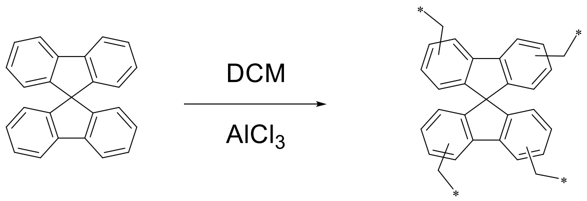

SBF (1.02 g, 3.22 mmol) and $AlCl_3$ (4.47 g, 33.5 mmol) were added to DCM (60 mL) and stirred at reflux for 24 hours under a nitrogen atmosphere. The solution was filtered, and the obtained powder washed with plenty of water and ethanol. The brown powder was washed sequentially via reflux with ethanol, chloroform, THF, acetone, and methanol, and finally dried in a vacuum oven at 100 ^{0}C for 20 hours. The SBF-network polymer (1.40 g, 3.80 mmol, 118% yield based on ideal structure. To be considered quantitative but the complete removal of the trapped gases/moisture was impossible) was analysed by IR spectroscopy, thermogravimetric analysis (TGA) and BET. SA_{BET}= 1604 m^2g^{-1}, CO_2 adsorption at 273 K/1 bar= 3.28 mmol g^{-1}, TGA: Thermal degradation commences at 255 $^{\circ}$C. FTIR-ATR (cm^{-1}): 906, 1172. 3060. ^{13}C NMR SS (101 MHz) δ 147.71, 139.99, 132.26, 127.02, 122.70, 72.66, 66.14, 54.95, 36.46, 16.66, 13.48.

PIM-HPB[4]

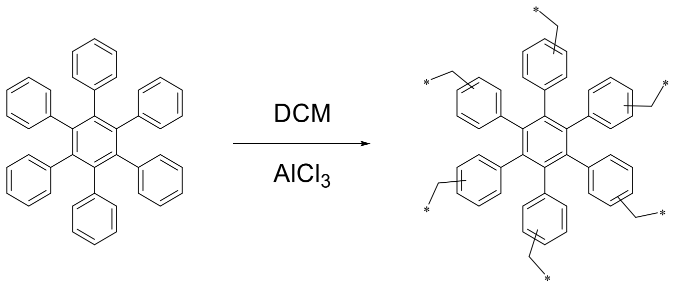

HPB (0.99 g, 1.85 mmol) and $AlCl_3$ (3.28 g, 24.59 mmol) were added to DCM (60 mL) and stirred at reflux for 24 hours under a nitrogen atmosphere. The solution was filtered, and the obtained powder washed with plenty of water and ethanol. The powder was washed sequentially via reflux with ethanol, chloroform, THF, acetone, and methanol, and finally dried in a vacuum oven at 100 ^{0}C for 20 hours. The HPB-network polymer (1.30 g, 2.12 mmol, 115% yield based on ideal structure. To be considered quantitative but the complete removal of the trapped gases/moisture was impossible) was analysed by IR spectroscopy, TGA, and BET. SA_{BET}= 1932 m^2g^{-1}, CO_2 adsorption at 273 K/1 bar= 3.12 mmol g^{-1}, TGA: Thermal degradation commences at 295 ˚C. FTIR-ATR (cm^{-1}): 1096, 1452, 2978. ^{13}C NMR SS (101 MHz) δ 136.69, 131.03, 126.72, 82.72, 72.49, 65.76, 54.44, 40.27, 34.15, 13.10.

General procedure for sulfonation of polymers- SBF-SO_3H and HPB-SO_3H

PIM-SBF (0.51 g, 1.38 mmol) or PIM-HPB (0.49 g, 0.79 mmol) was added to concentrated H_2SO_4 (20 mL) and stirred for 30 minutes, then warmed to 60 ^{o}C and stirred for more 7 hours under a nitrogen atmosphere. The solution was cooled to room temperature and poured over ice, filtered and the obtained solid was washed with plenty of water until a neutral pH of the residue was obtained. The resulting dark powder was refluxed in ethanol and methanol, filtered off and dried in a vacuum oven at 100 ^{o}C for 20 hours. The polymers, PIM-SBF-SO_3H and PIM-HPB-SO_3H, were analysed by IR spectroscopy, TGA, and BET.

PIM-SBF-HSO_3 (0.52 g, 0.76 mmol, 64%): SA_{BET}= 1063 m^2g^{-1}, CO_2 adsorption at 273 K/1 bar= 3.47 mmol g^{-1}, TGA: Thermal degradation commences at 167 ˚C. FTIR-ATR (cm^{-1}): 1038, 1190, 2980, 3450. ^{13}C NMR SS (101 MHz) δ 184.97, 140.47, 133.24, 128.25, 66.50, 57.09, 38.78, 19.11.

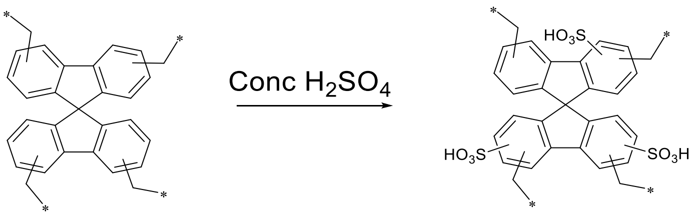

PIM-HPB-HSO₃ (0.57 g, 0.52 mmol, 69%): SA_{BET}= 1390 m^2g^{-1}, CO_2 adsorption at 273 K/1 bar= 2.92 mmol g^{-1}, TGA: Thermal degradation commences at 165 ˚C. FTIR-ATR (cm^{-1}): 1039, 1175, 2980, 3420. ^{13}C NMR SS (101 MHz) δ 255.64, 235.97, 192.27, 135.92, 131.84, 72.44, 55.35, 34.28, 14.61.

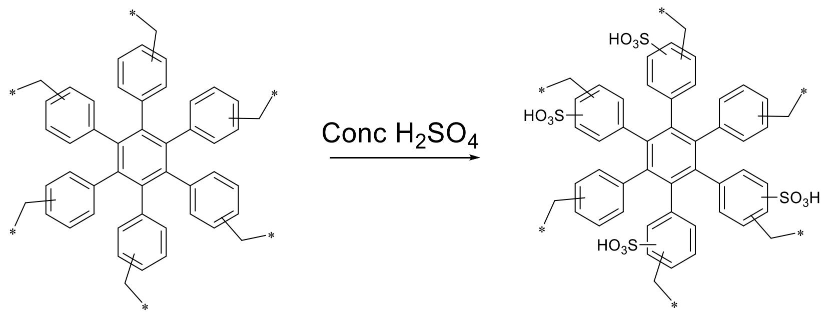

General procedure for nitration of polymers- PIM-SBF-NO_2 and PIM-HPB-NO_2

Under a nitrogen atmosphere, H_2SO_4 (50 mL) was cooled in an ice bath for 20 minutes before PIM-SBF (0.49 g, 1.33 mmol) or PIM-HPB (0.50 g, 0.82 mmol) or PIM-TPB (0.50 g, 1.44 mmol) or PIM-TRIP (0.50 g, 1.70 mmol) were added. Concentrated HNO_3 (0.5 mL, 70%) was added dropwise and the solution was then stirred for 2 hours in an ice bath. The resulting solution was poured over ice, stirred for 30 minutes, filtered and the obtained solid was washed with plenty of water until a neutral pH of the residue was obtained. The resulting dark powder was n refluxed in ethanol and methanol, filtered and dried in a vacuum oven at 100 °C for 20 hours. The polymers, PIM-SBF-NO_2 and PIM-HPB-NO_2, were analysed by IR spectroscopy, TGA, and BET.

PIM-SBF-NO_2 (0.55 g, 1.00 mmol, 83%): SA_{BET}= 909 m^2g^{-1}, CO_2 adsorption at 273 K/1 bar= 3.32 mmol g^{-1}, TGA: Thermal degradation commences at 230 ˚C. FTIR-ATR (cm^{-1}): 1344, 1538, 3051. ^{13}C NMR SS (101 MHz) δ 192.17, 182.57, 145.51, 134.90, 125.58, 66.61, 56.04, 53.18, 36.63, 14.21.

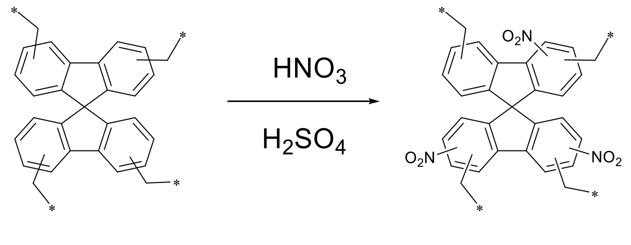

PIM-HPB- NO_2 (0.58 g, 0.66 mmol, 69%): SA_{BET}= 1286 m^2g^{-1}, CO_2 adsorption at 273 K/1 bar= 3.12 mmol g^{-1}, TGA: Thermal degradation commences at 216 ˚C. FTIR-ATR (cm^{-1}): 1349, 1536, 3023. ^{13}C NMR SS (101 MHz) δ 186.18, 133.90, 70.27, 57.00, 34.32, 14.75.

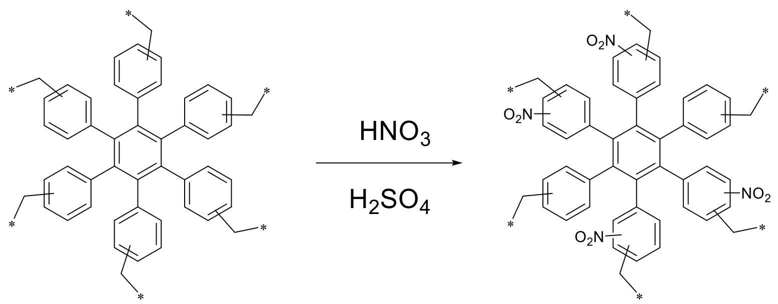

PIM-TPB- NO_2 (0.60 g, 1.15 mmol, 80%). SA_{BET}= 950 m^2g^{-1}, CO_2 adsorption at 273 K/1 bar= 5.13 mmol g^{-1}, TGA: Thermal degradation commences at 180 ˚C. FTIR-ATR (cm^{-1}): 1048, 1298, 1676, 2972. ^{13}C NMR SS (101 MHz) δ 182.79, 162.93, 133.65, 72.66, 55.09, 35.12, 30.24, 16.50.

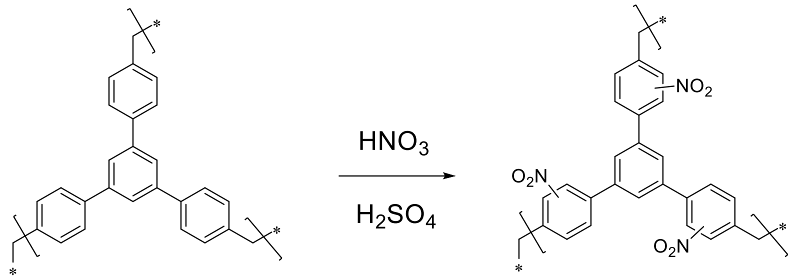

PIM-TRIP- NO_2 (0.62, 1.45 mmol, 85%) SA_{BET}= 975 m^2g^{-1}, CO_2 adsorption at 273 K/1 bar= 4.87 mmol g^{-1}, TGA: Thermal degradation commences at 195 ˚C. FTIR-ATR (cm^{-1}): 1046, 1295, 1675, 2992. ^{13}C NMR SS (101 MHz) δ 182.37, 163.14, 145.72, 132.16, 73.49, 49.61, 35.59, 30.45, 14.82.

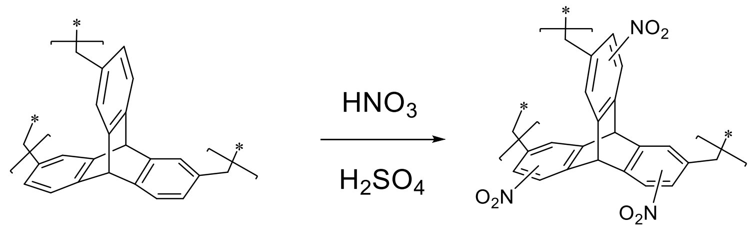

General procedure for amination of polymers: SBF-NH_2 and HPB-NH_2

PIM-SBF-NO_2 (0.30 g, 0.61 mmol) or PIM-HPB-NO_2 (0.30 g, 0.42 mmol) or PIM-TPB-NO_2 (0.30 g, 0.625 mmol) or PIM-TRIP-NO_2 (0.30 g, 0.701 mmol) were added to a solution of methanol and water (30 mL each), and the solution was degassed by a flow of nitrogen for 20 minutes. Sodium dithionite (4.01 g, 23 mmol) was added, and the solution stirred for 24 hours at 75 °C under a nitrogen atmosphere. The solution was filtered, and the resulting powder was washed with hot water. The powder was dispersed in 75 °C water (60 mL) and stirred for 30 minutes, then filtered and washed again with hot water. After being dried under reduced pressure, the powder was suspended in 4M HCl (60 mL) and stirred for 1 hour, then filtered and washed with plenty of water until a neutral pH of the residue was obtained. The resulting powder was refluxed in methanol, filtered and dried in a vacuum oven at 100 °C for 20 hours. The polymers, PIM-SBF-NH_2 and PIM-HPB-NH_2, were analysed by IR spectroscopy, TGA, and BET.

PIM-SBF-NH₂ (0.15 g, 0.40 mmol, 64%): SA_{BET}= 669 m^2g^{-1}, CO_2 adsorption at 273 K/1 bar= 2.9 mmol g^{-1}, TGA: Thermal degradation commences at 190 °C. FTIR-ATR (cm^{-1}): 1080, 1595, 3000, 3264. ^{13}C NMR SS (101 MHz) δ 254.48, 182.63, 170.09, 140.98, 134.43, 126.43, 66.62, 53.60, 31.37, 13.51.

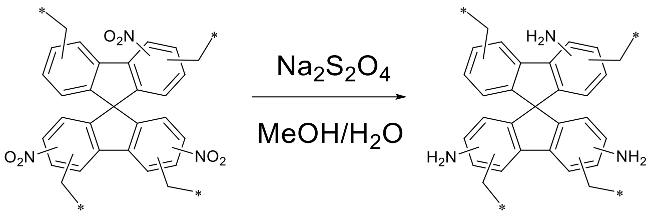

PIM-HPB-NH₂ (0.22 g, 0.37 mmol, 88%): SA_{BET}= 997 m^2g^{-1}, CO_2 adsorption at 273 K/1 bar= 2.80 mmol g^{-1}, TGA: Thermal degradation commences at 161 °C. FTIR-ATR (cm^{-1}): 1074, 1196, 1602, 2950, 3348. ^{13}C NMR SS (101 MHz) δ 226.77, 186.88, 133.37, 71.30, 53.53, 34.06, 14.87.

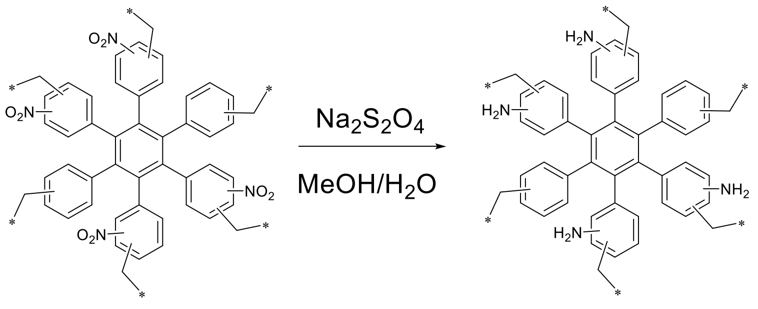

PIM-TPB-NH₂ (0.21 g, 0.53 mmol, 86%): SA_{BET}= 710 m^2g^{-1}, CO_2 adsorption at 273 K/1 bar= 4.45 mmol g^{-1}, TGA: Thermal degradation commences at 200 °C. FTIR-ATR (cm^{-1}): 1035, 1280, 1610, 2980, 3436. ^{13}C NMR SS (101 MHz) δ 184.28, 164.95, 133.26, 73.97, 51.34, 34.87, 15.58.

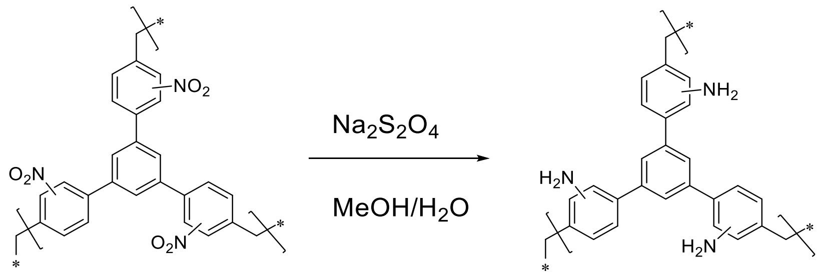

PIM-TRIP-NH₂ (0.18 g, 0.53 mmol, 76%): SA_{BET}= 997 m^2g^{-1}, CO_2 adsorption at 273 K/1 bar= 2.80 mmol g^{-1}, TGA: Thermal degradation commences at 221 °C. FTIR-ATR (cm^{-1}): 1046, 1200, 1594, 2950, 3405. ^{13}C NMR SS (101 MHz) δ 183.69, 163.22, 131.93, 72.26, 47.55, 35.46, 30.47, 14.62.

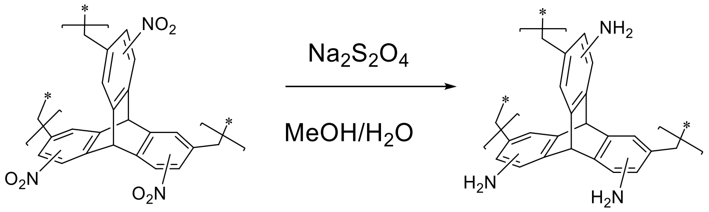

4. Selectivity CO_2/N_2

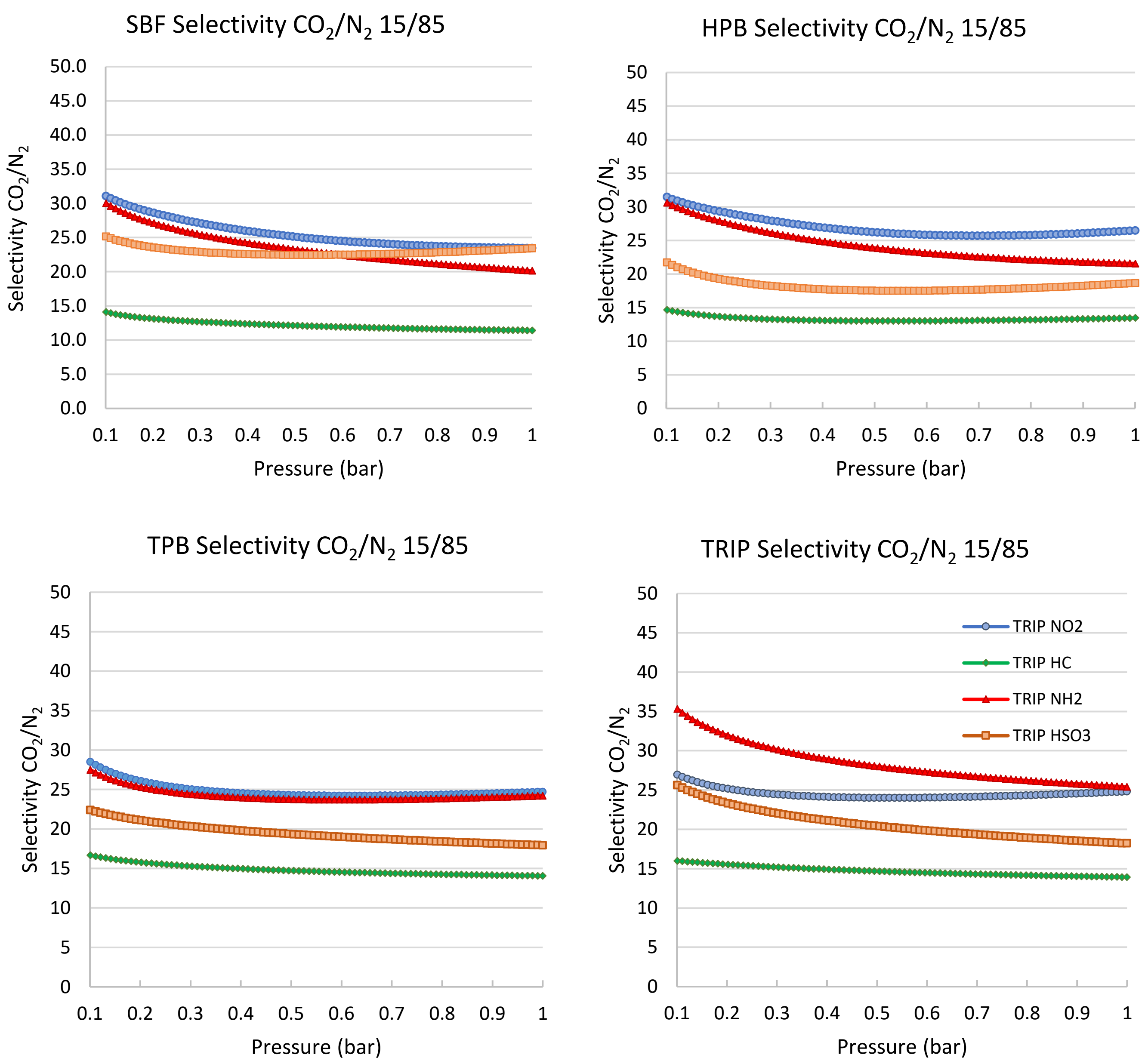

Figure SI1. Selectivity CO_2/N_2

5. Selectivity CO_2/CH_4

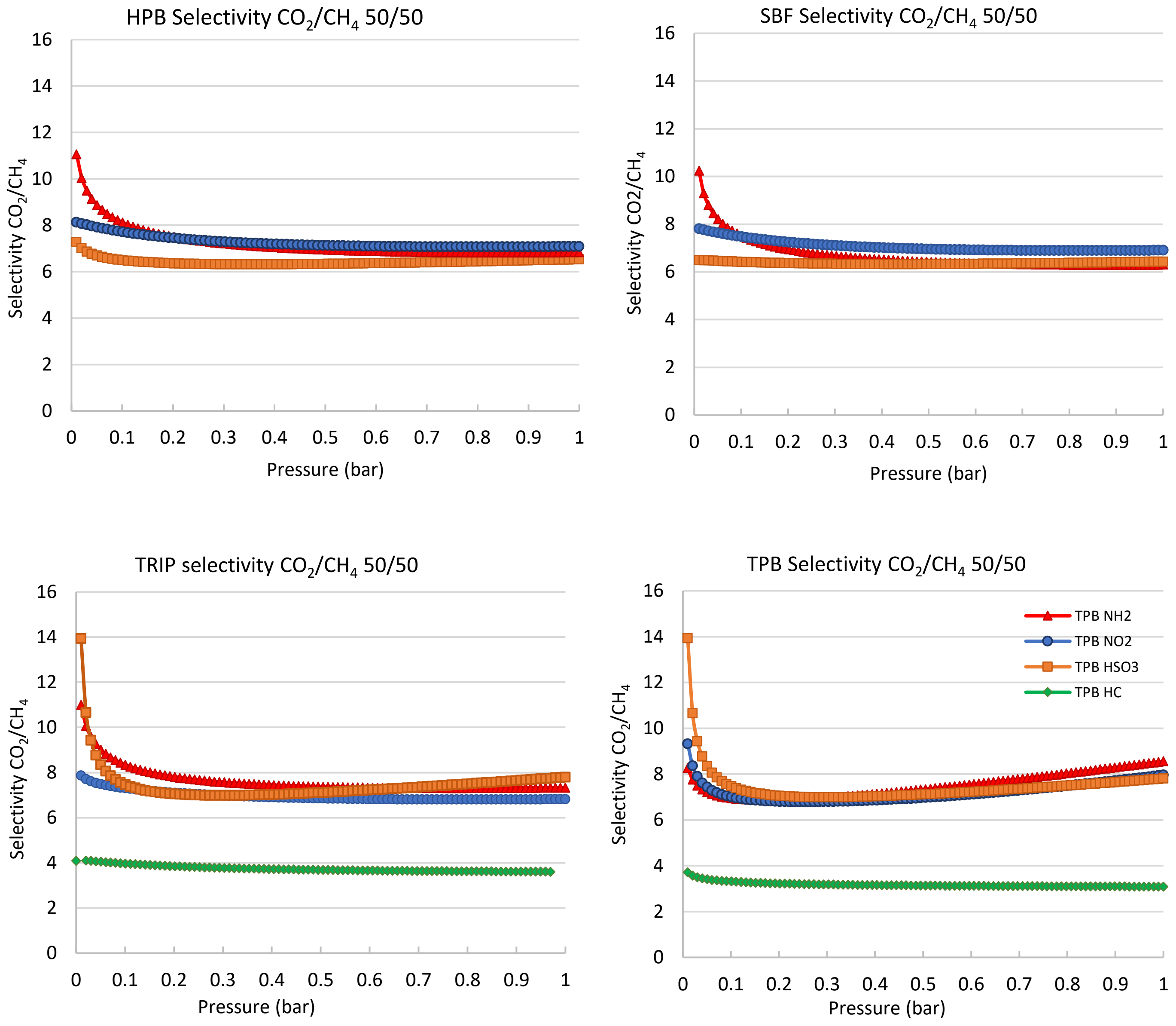

Figure SI2. Selectivity CO_2/CH_4

6. Isotherms

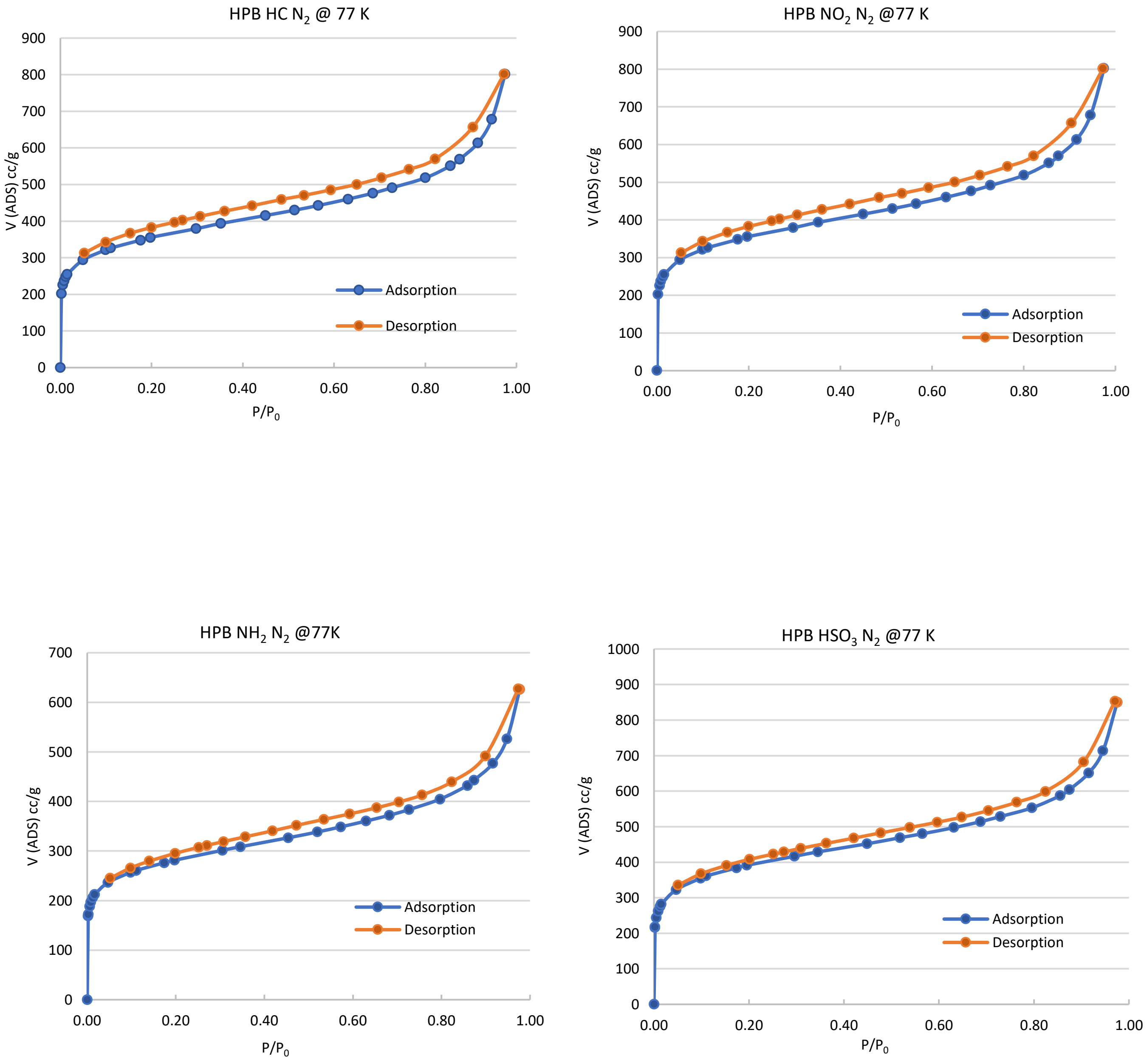

Figure SI3. Nitrogen adsorption **i**sotherms for HPB-PIMs

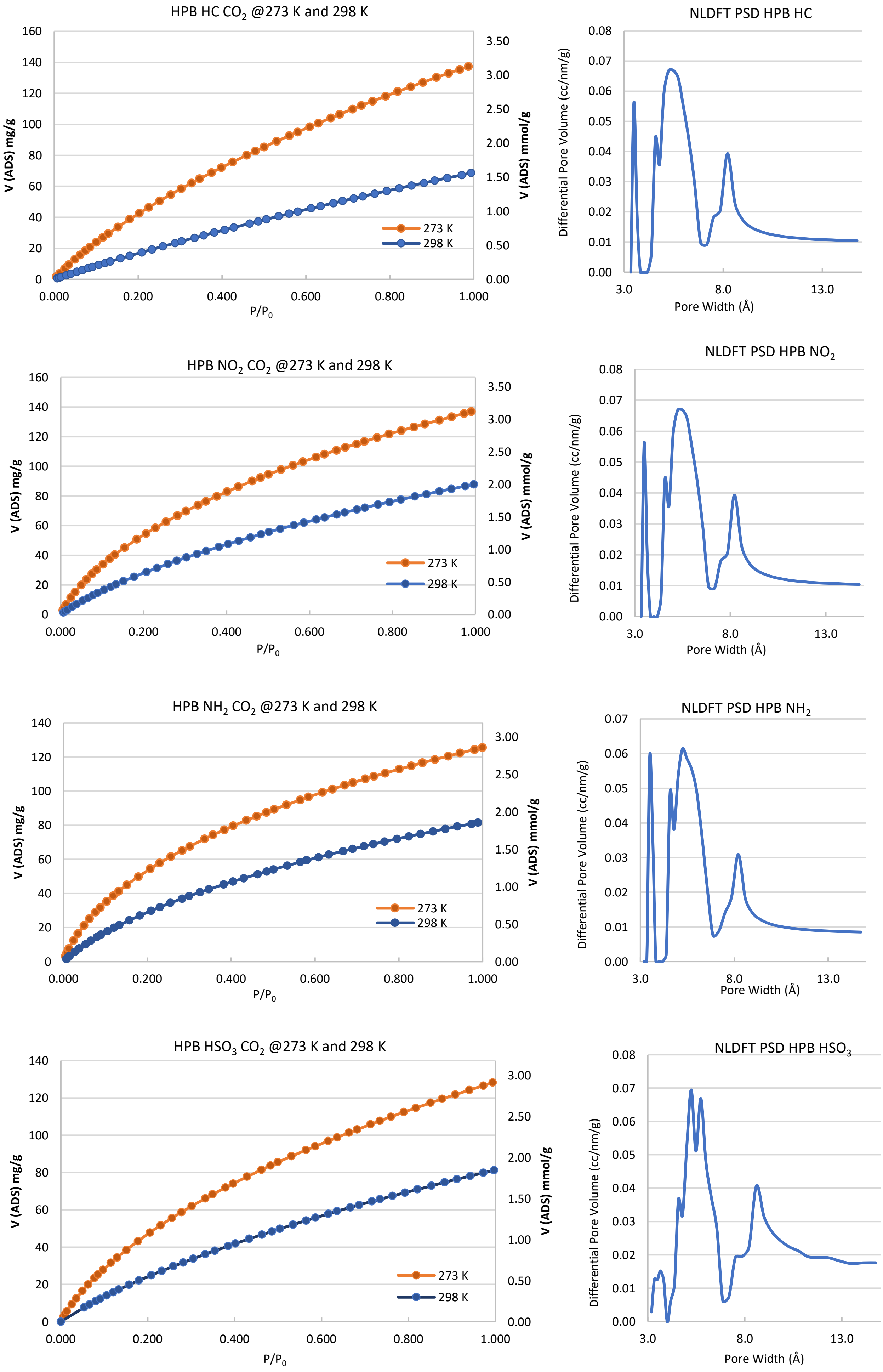

Figure SI4. CO_2 adsorption **i**sotherms for HPB-PIMs and NLDFT pore size distributions

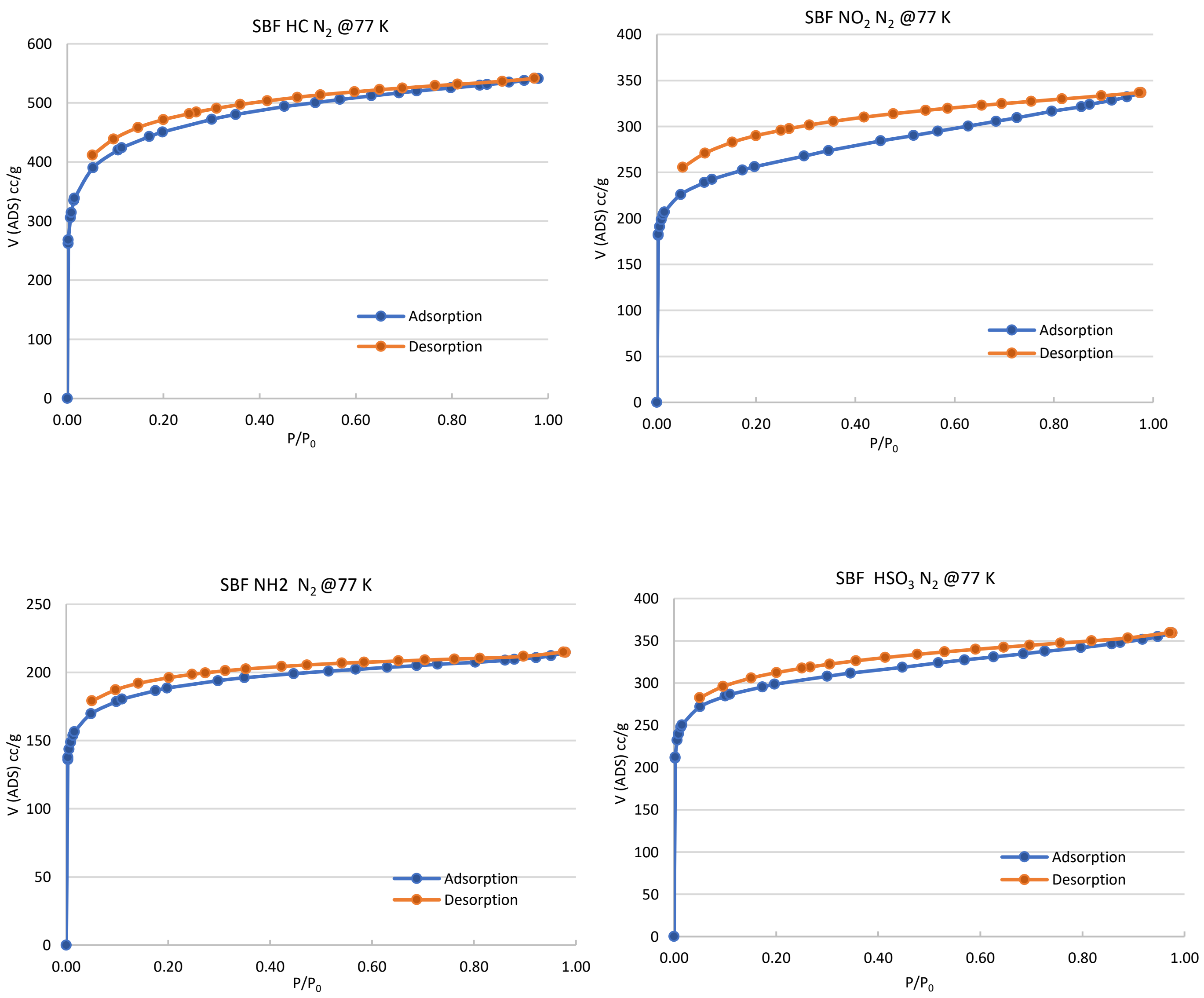

Figure SI5. Nitrogen adsorption **i**sotherms for SBF-PIMs

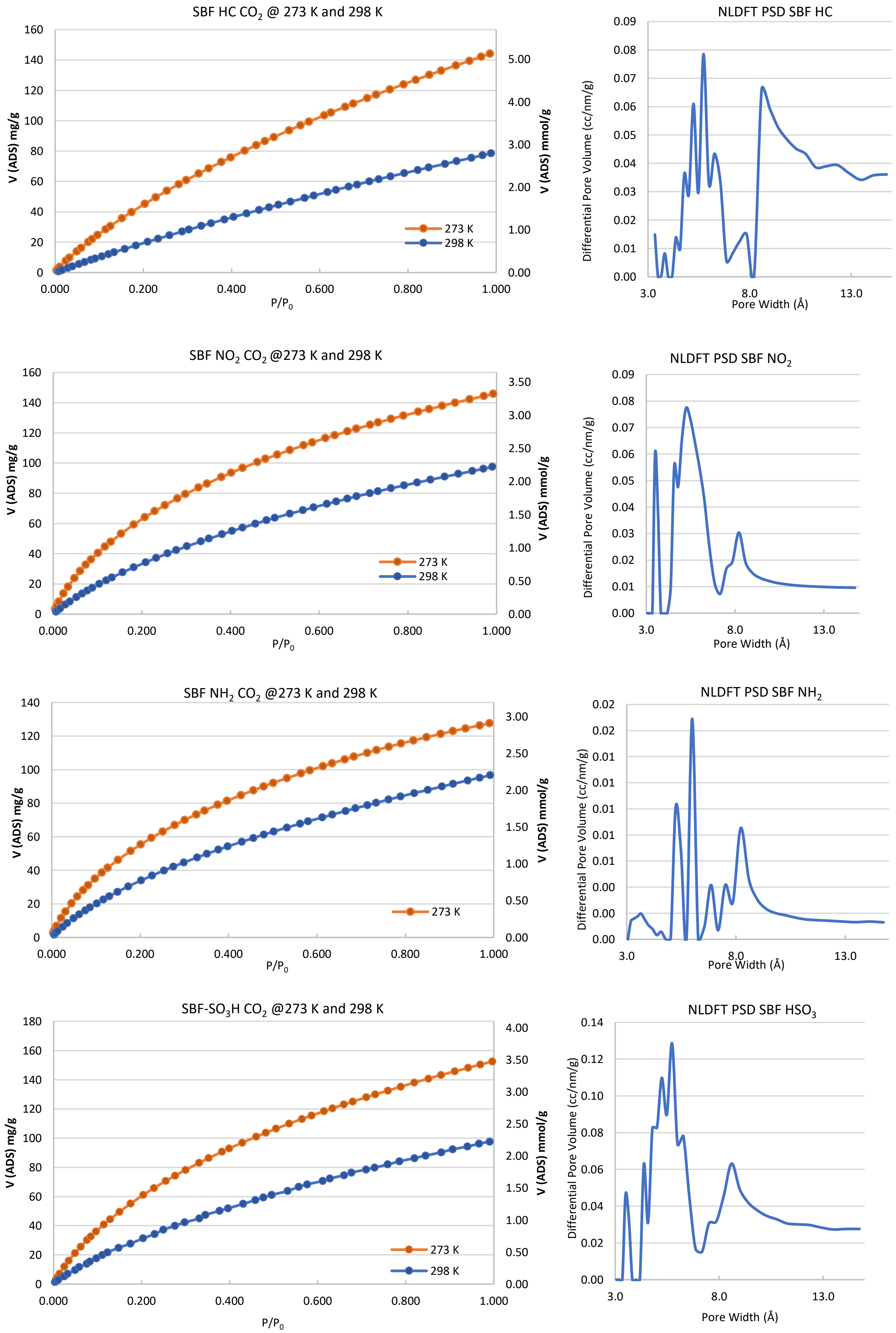

Figure SI6. CO_2 adsorption **i**sotherms for SBF-PIMs and NLDFT pore size distributions

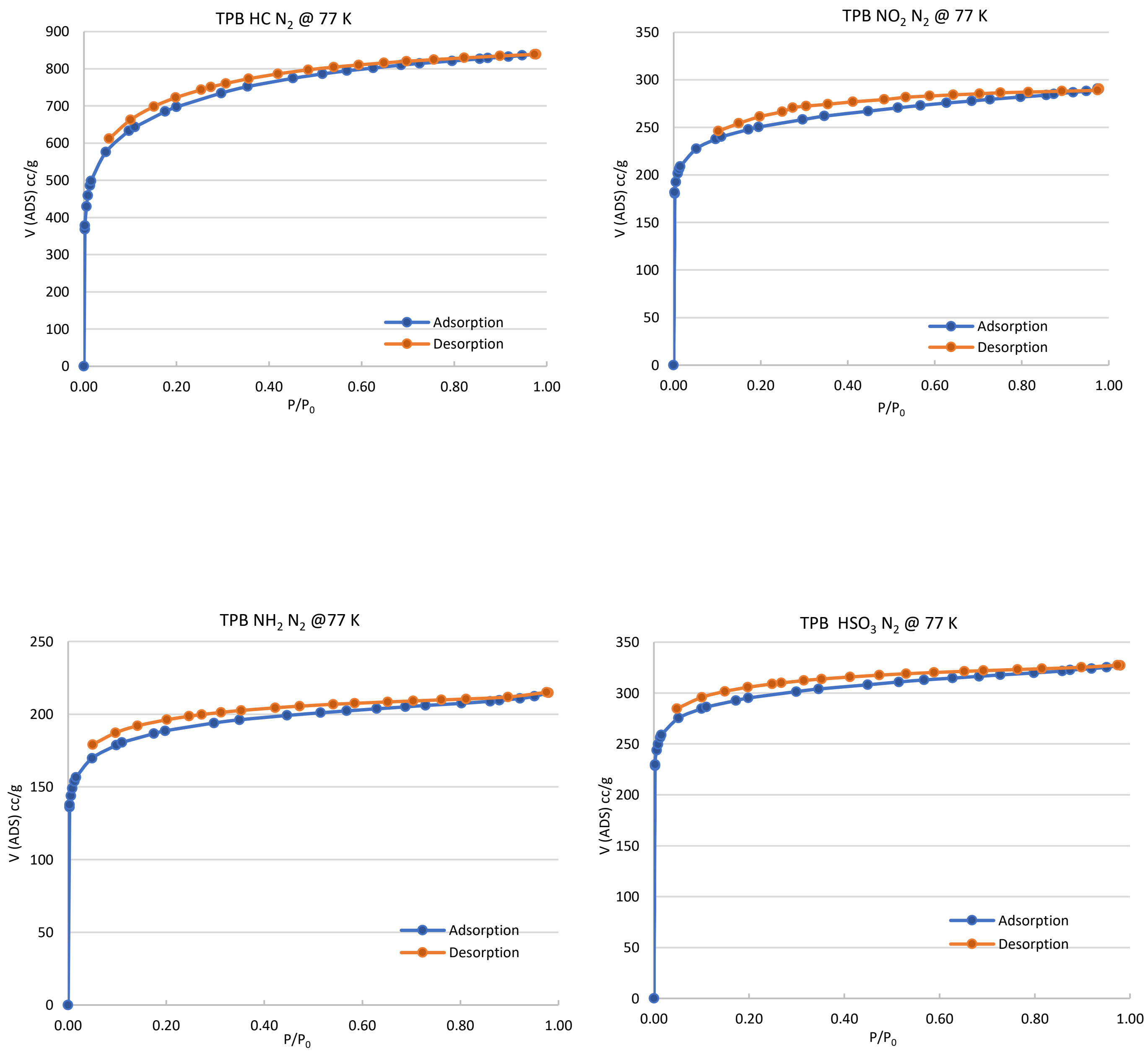

Figure SI7. Nitrogen adsorption **i**sotherms for TPB-PIMs

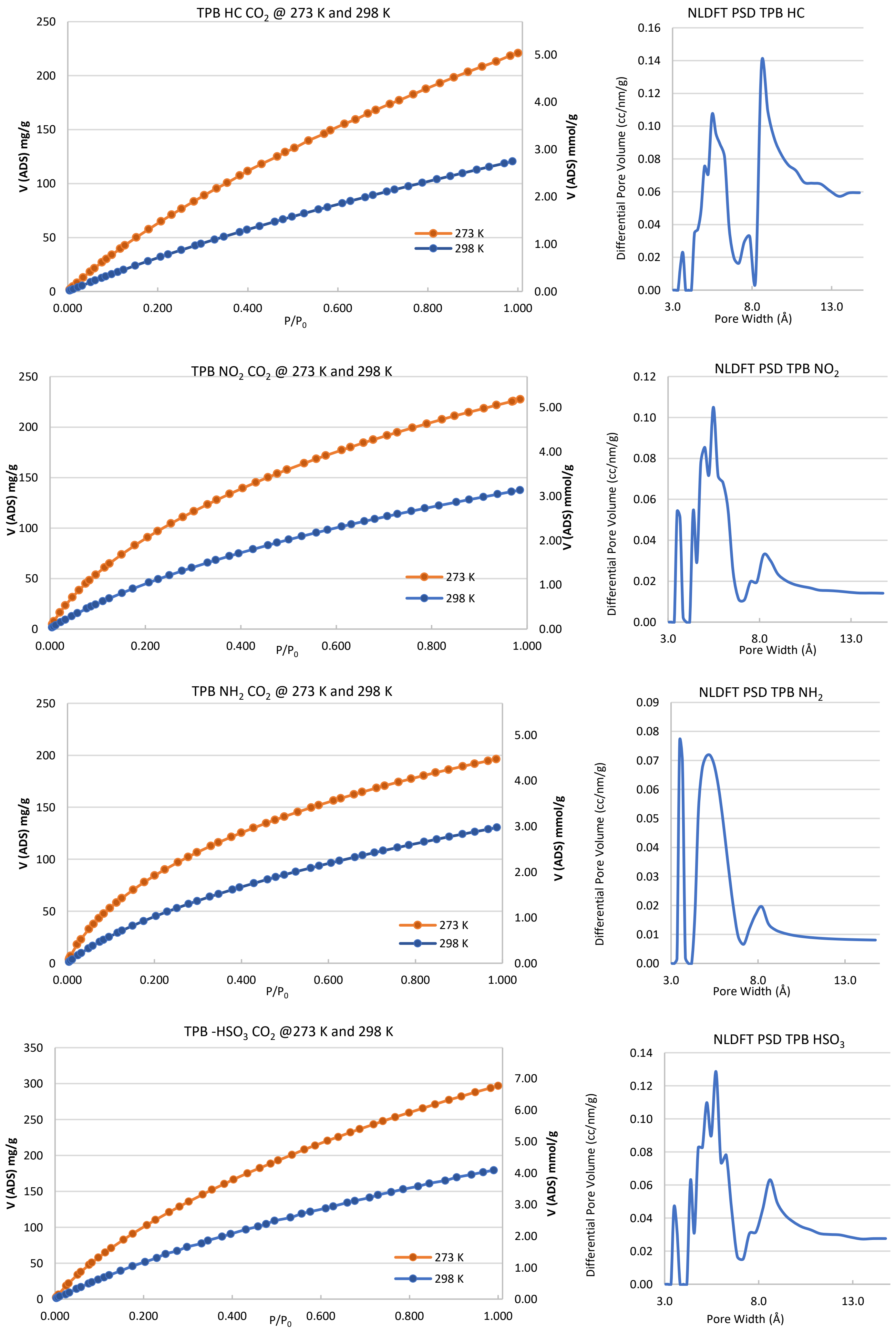

Figure SI8. CO_2 adsorption **i**sotherms for TPB-PIMs and NLDFT pore size distributions

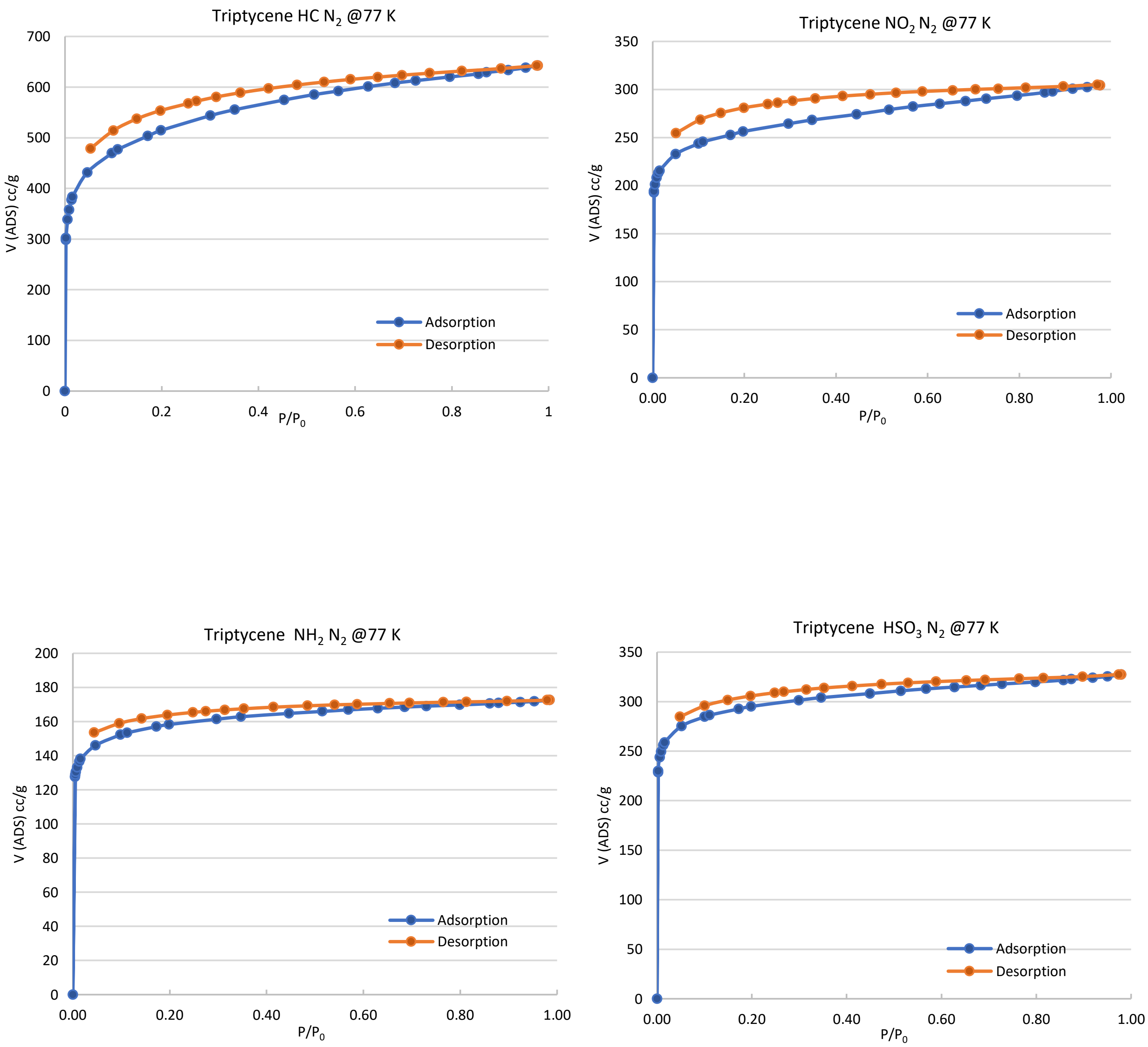

Figure SI9. Nitrogen adsorption **i**sotherms for TRIP-PIMs

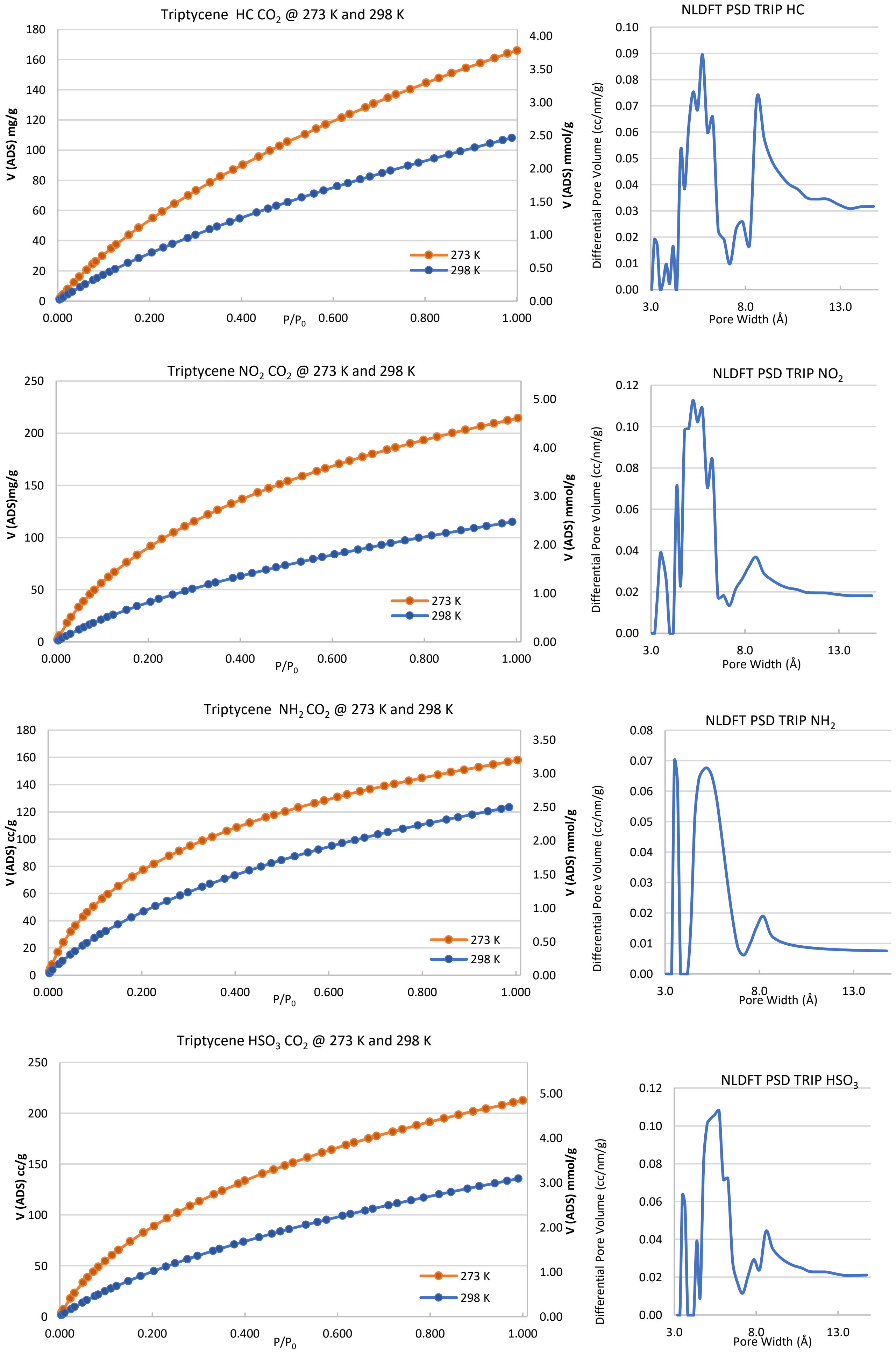

Figure SI10. CO_2 adsorption **i**sotherms for TRIP-PIMs and NLDFT pore size distributions

7. Heats of adsorption

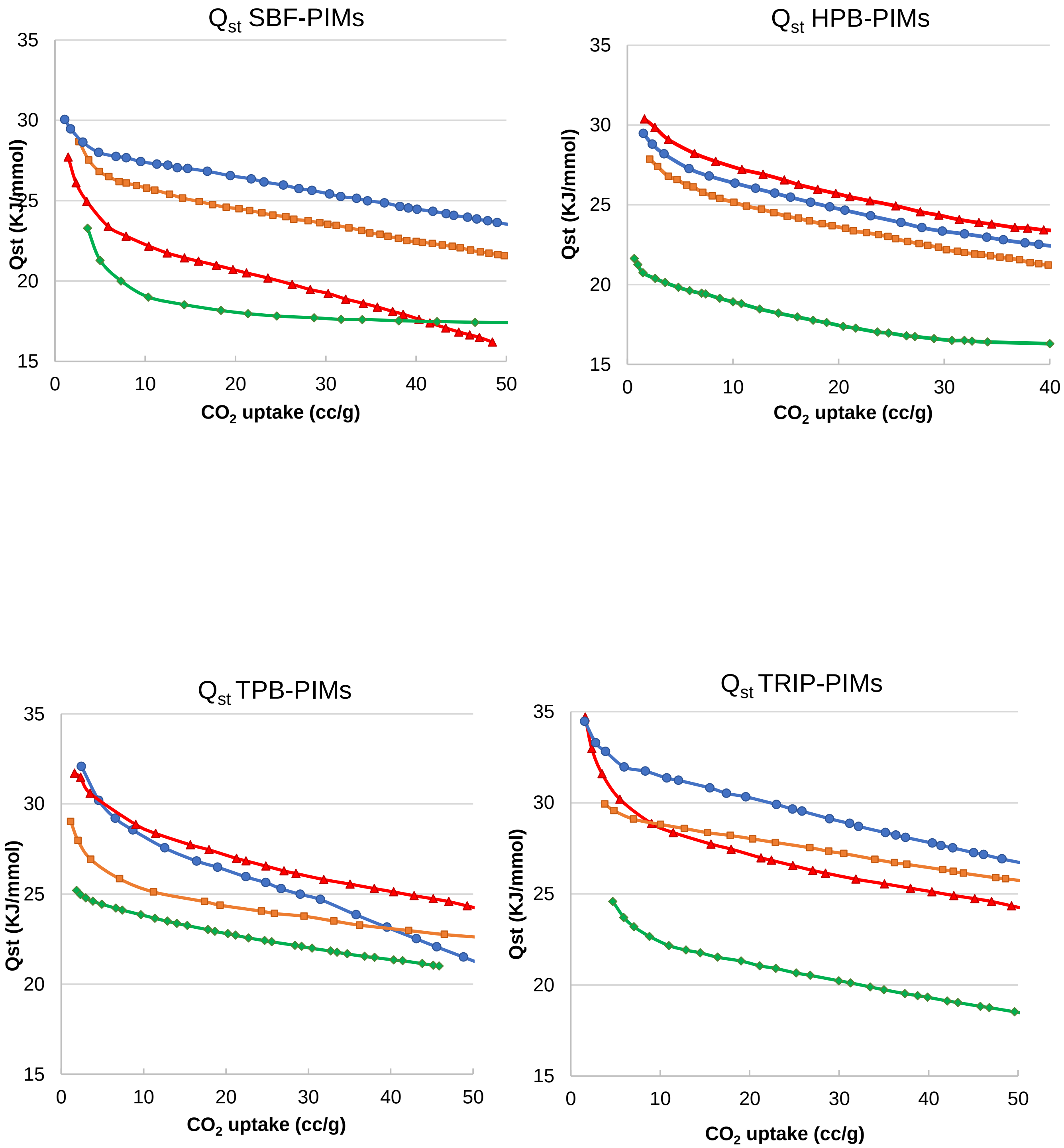

Figure SI11. Heats of adsorption for PIMs.

8. FT-IRs

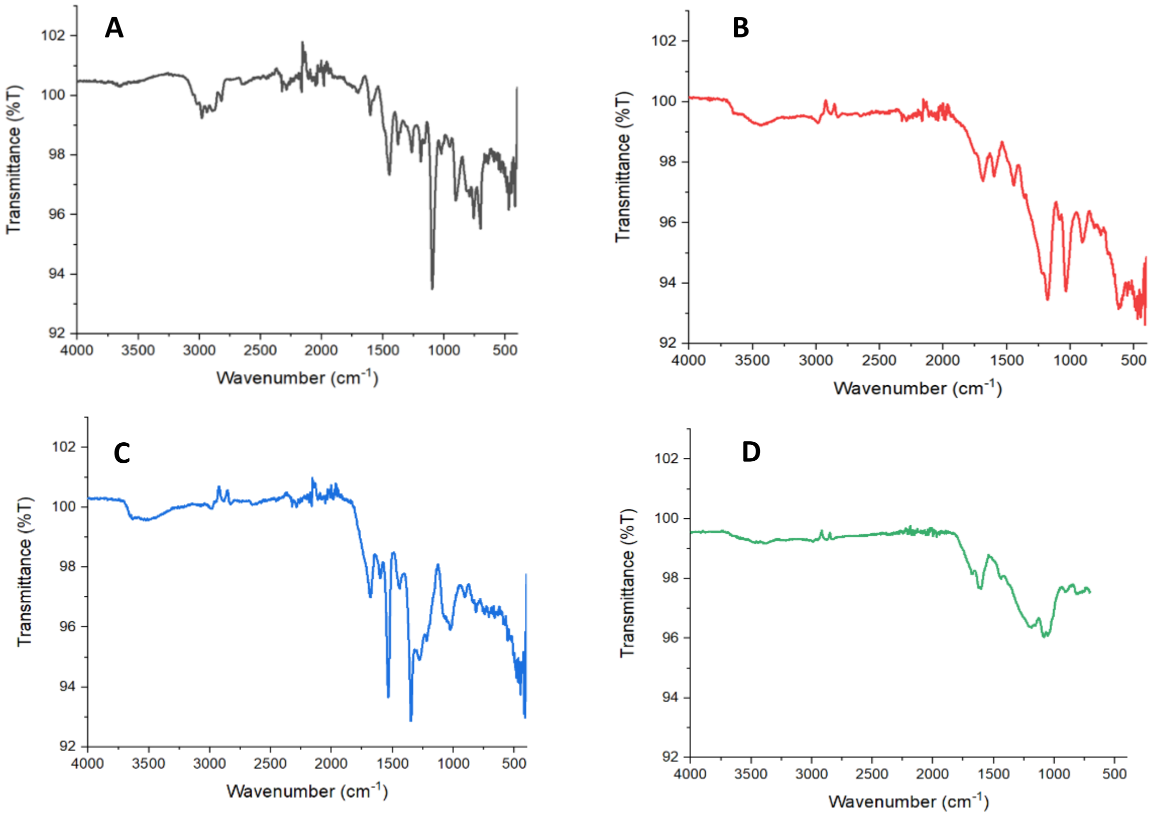

Figure SI12. FT-IR spectra of **HPB-PIMs** polymers. **A**- HC, **B**- -HSO_3, **C**- NO_2, **D**- NH_2.

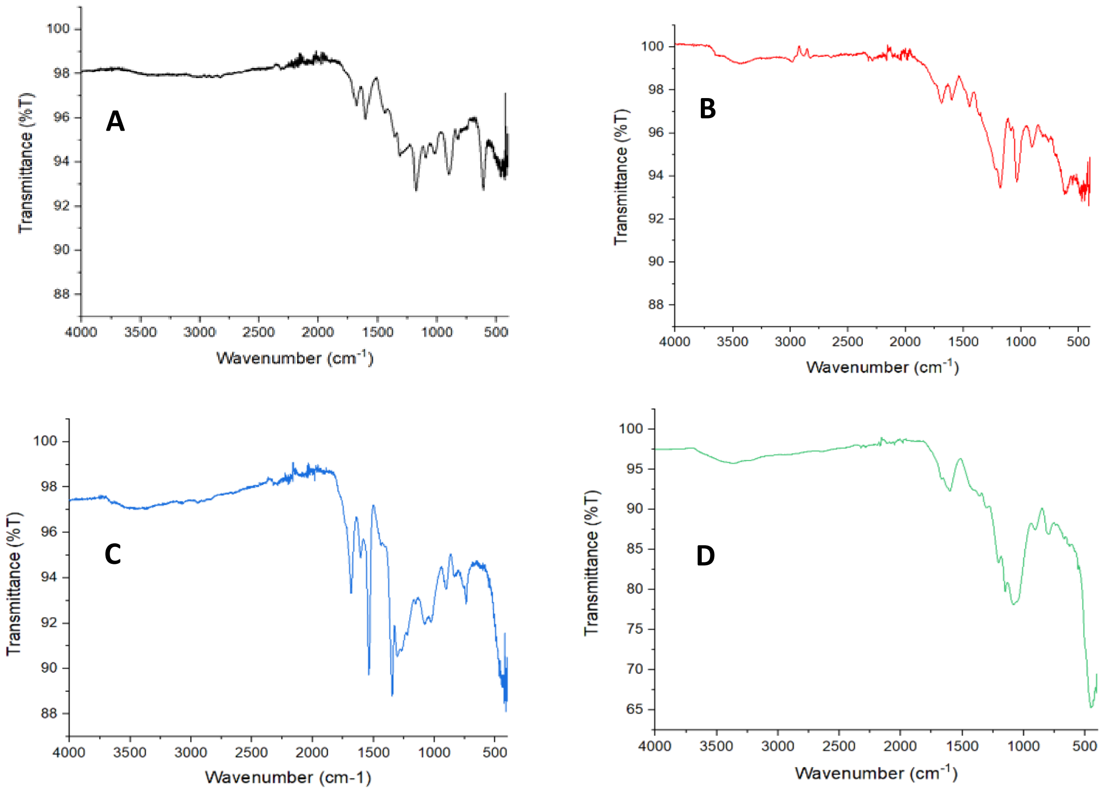

Figure SI13. FT-IR spectra of **SBF-PIMs** polymers. **A**- HC, **B**- -HSO_3, **C**- NO_2, **D**- NH_2.

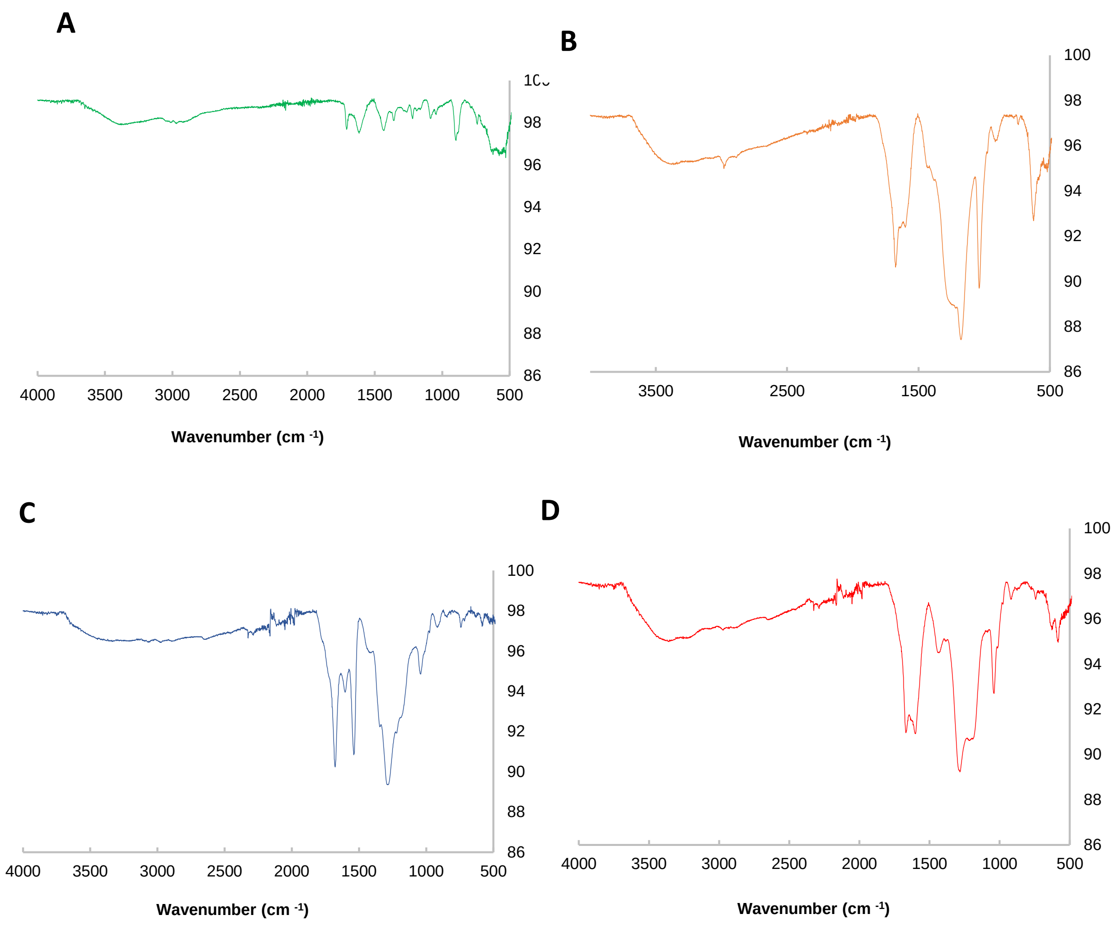

Figure SI14. FT-IR spectra of **TRIP-PIMs** polymers. **A**- HC, **B**- -HSO_3, **C**- NO_2, **D**- NH_2.

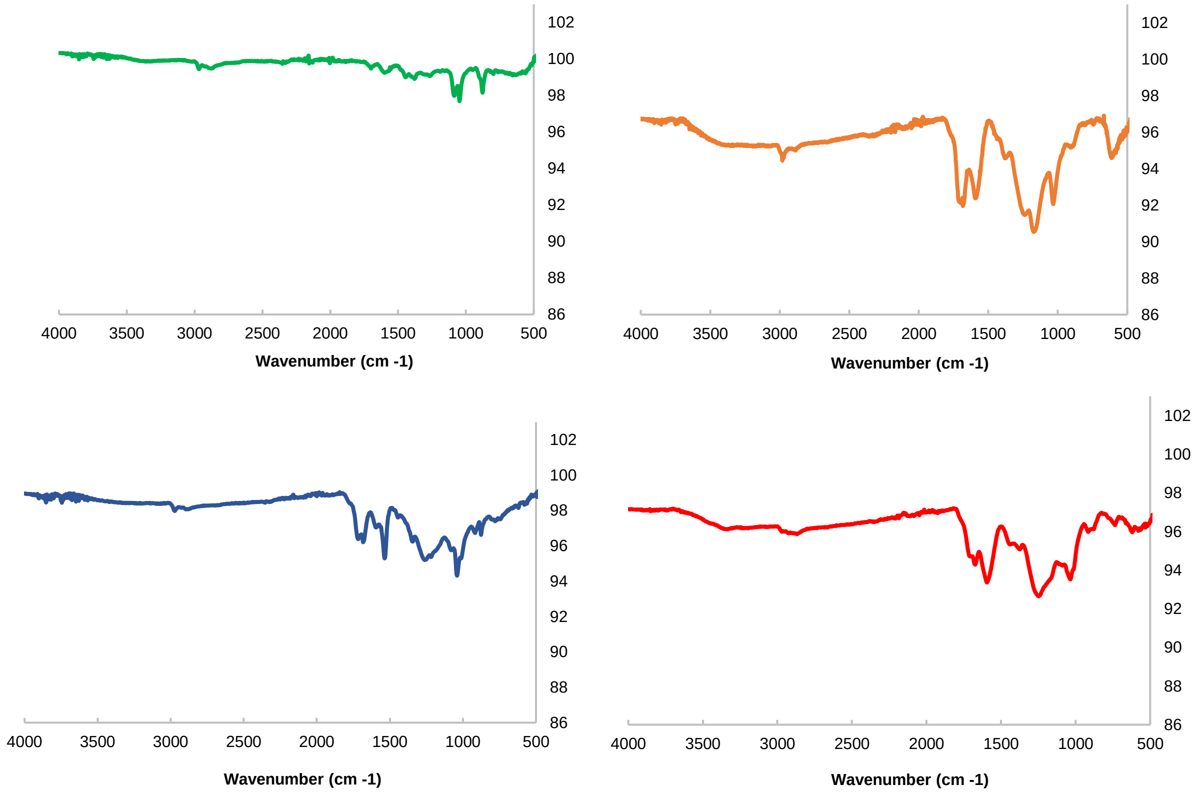

Figure SI15. FT-IR spectra of **TPB-PIMs** polymers. **A**- HC, **B**- -HSO_3, **C**- NO_2, **D**- NH_2.

9. Solid state ^{13}C NMRs

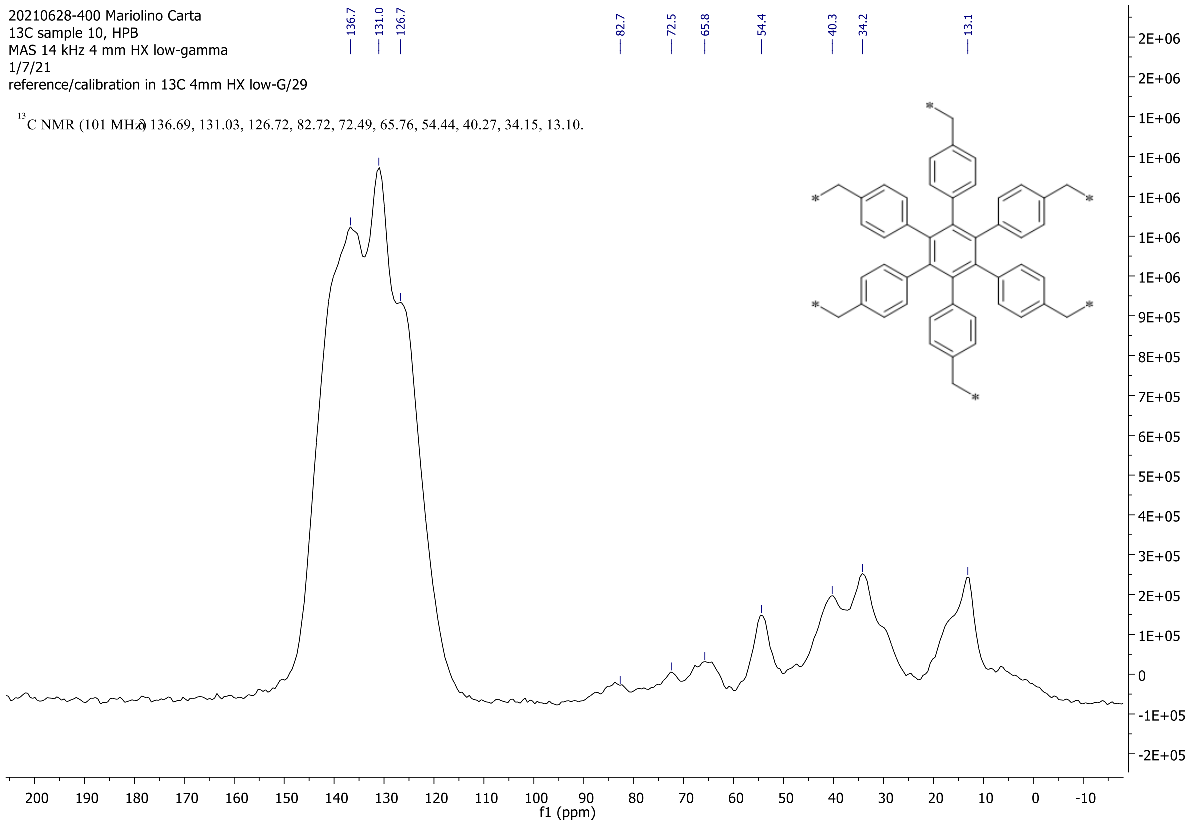

Figure SI16. ^{13}C solid state NMR for HPB-HC

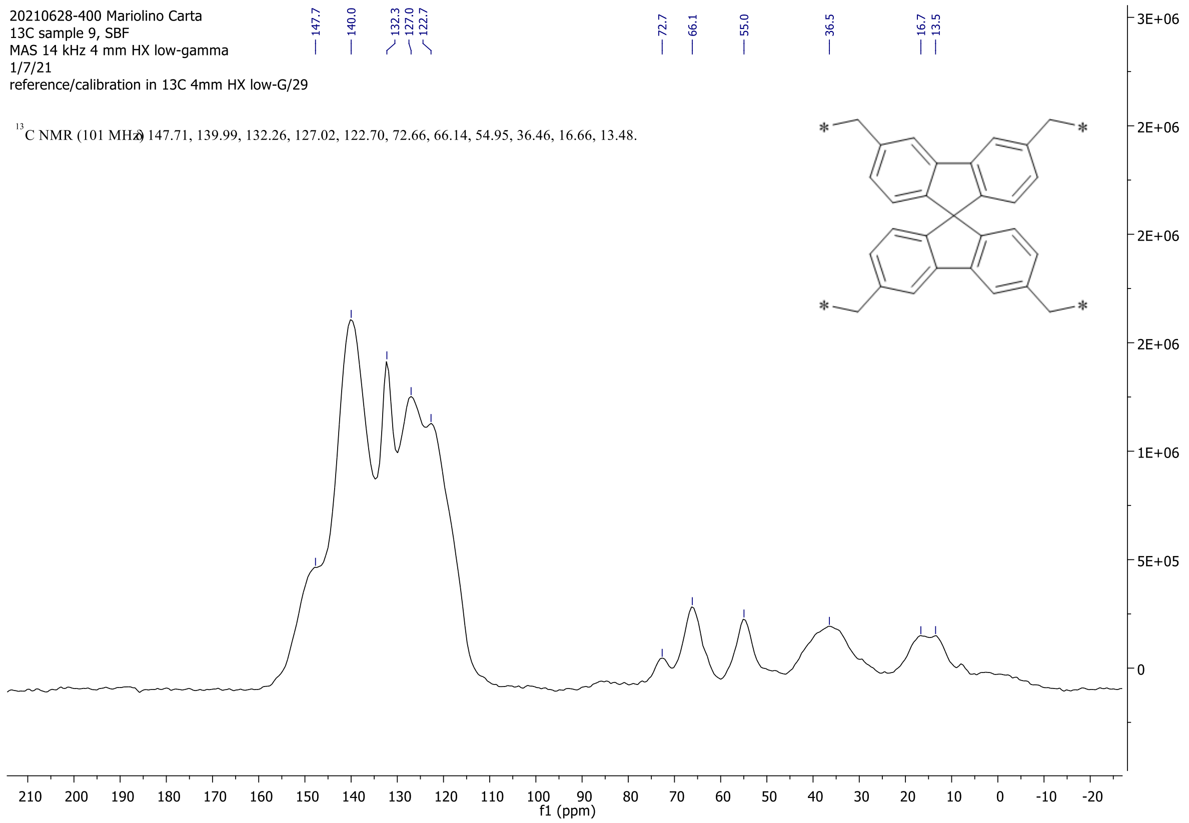

Figure SI17. ^{13}C solid state NMR for SBF-HC

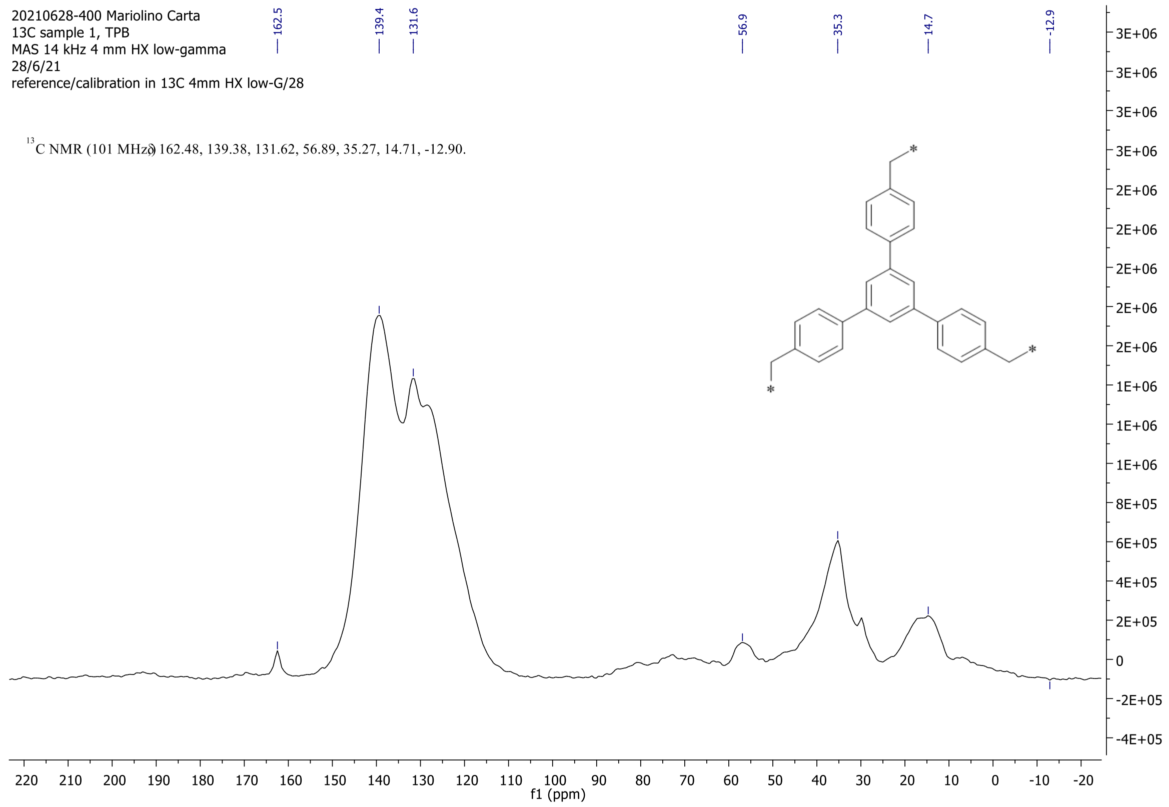

Figure SI18. ^{13}C solid state NMR for TPB-HC

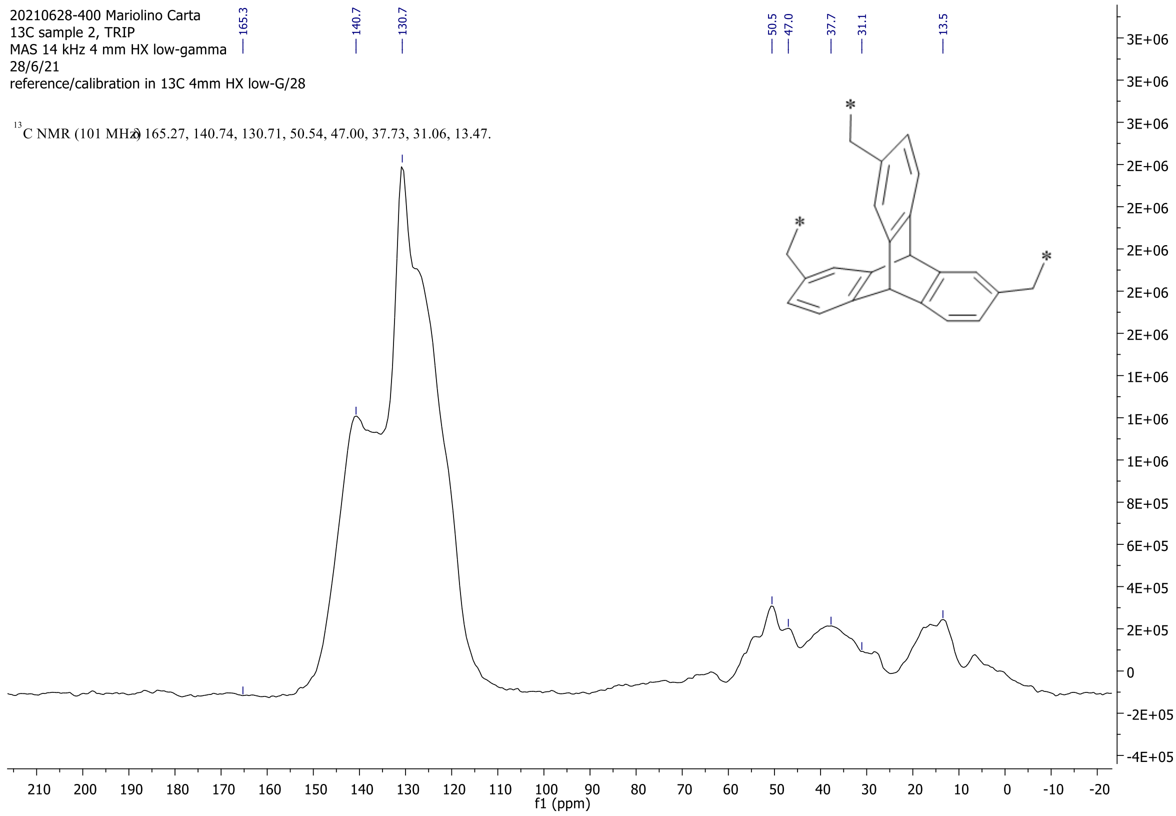

Figure SI19. ^{13}C solid state NMR for TRIP-HC

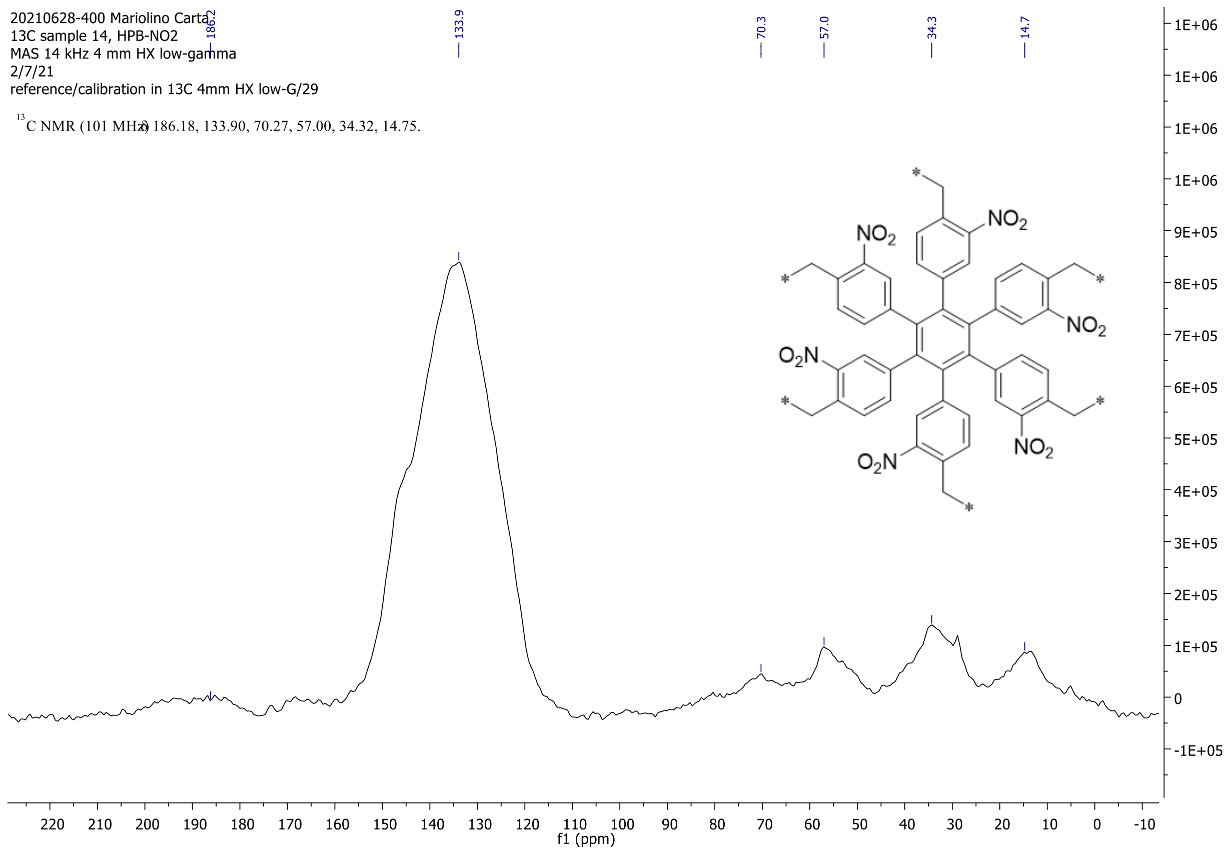

Figure SI20. ^{13}C solid state NMR for HPB-NO_2

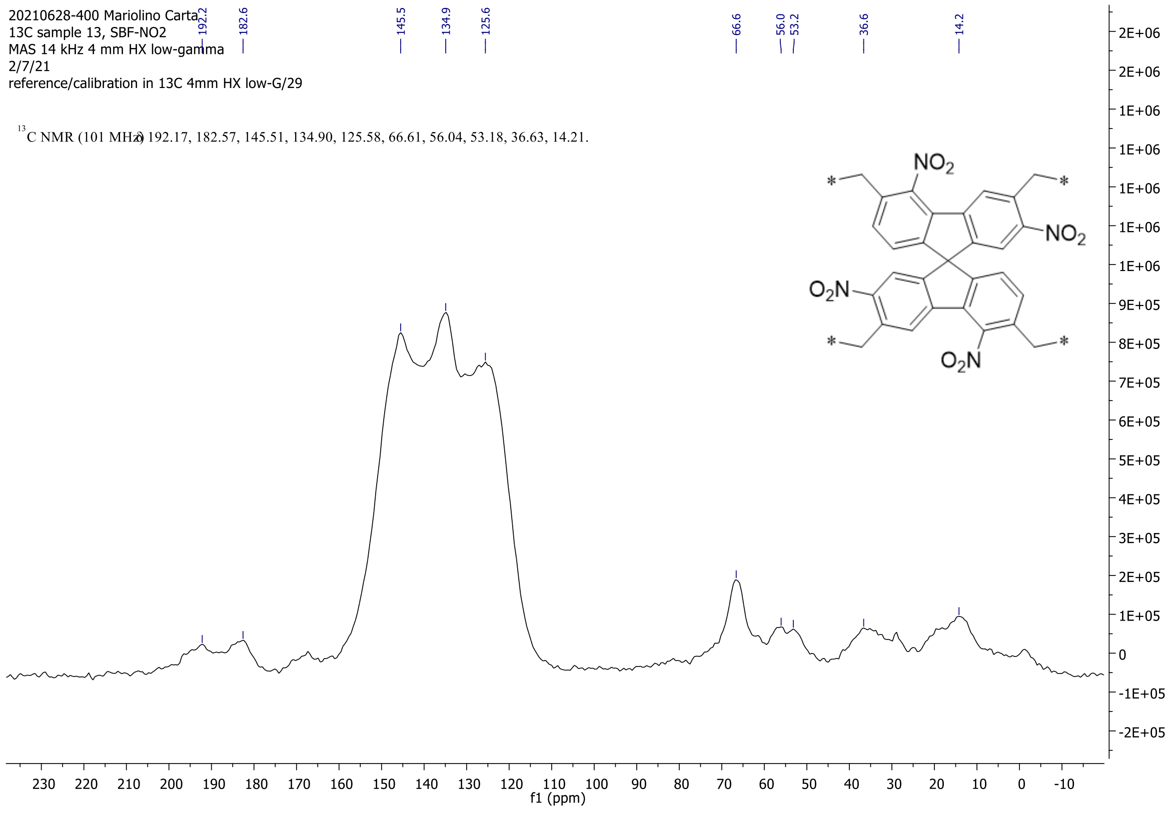

Figure SI21. ^{13}C solid state NMR for SBF-NO_2

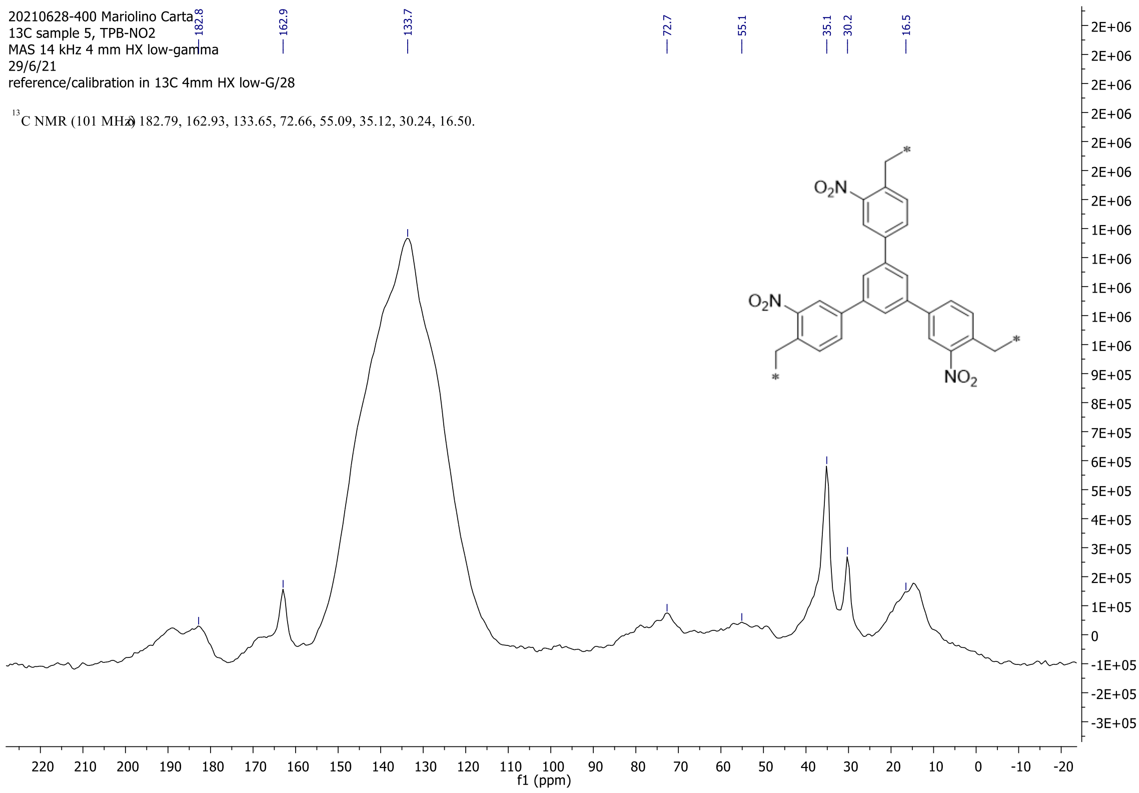

Figure SI22. ^{13}C solid state NMR for TPB-NO_2

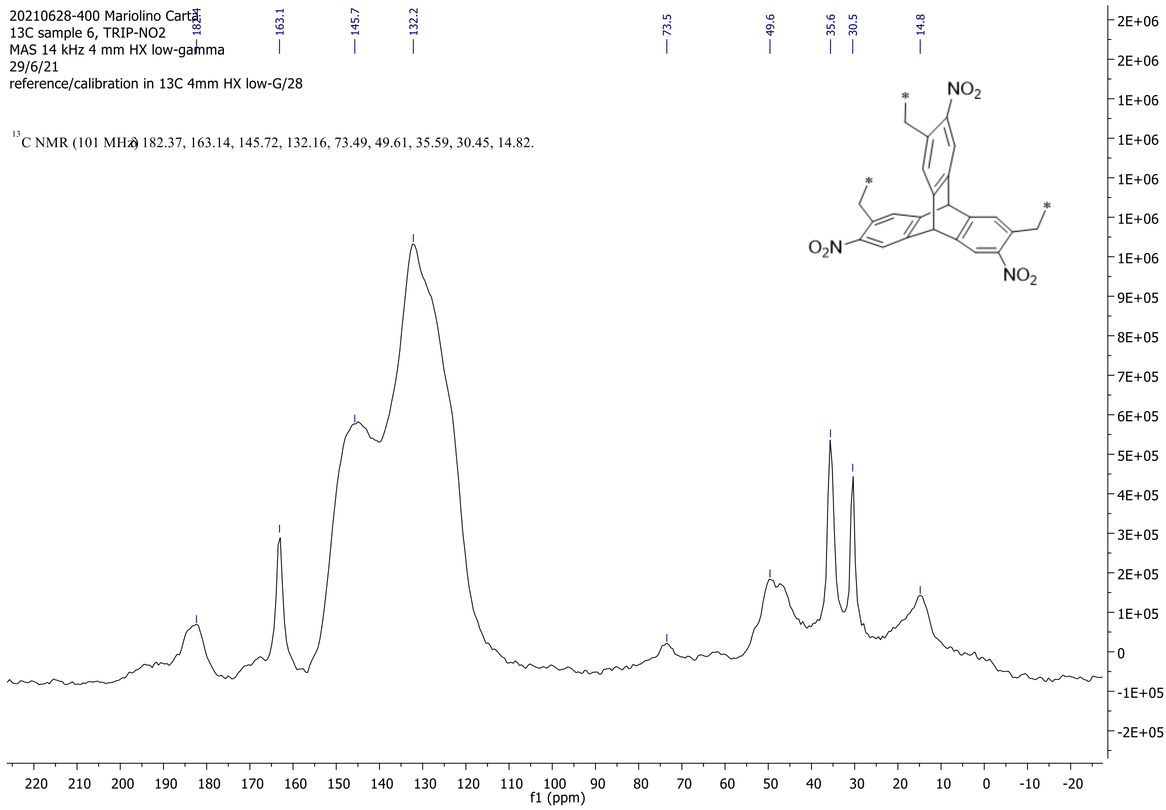

Figure SI23. ^{13}C solid state NMR for TRIP-NO_2

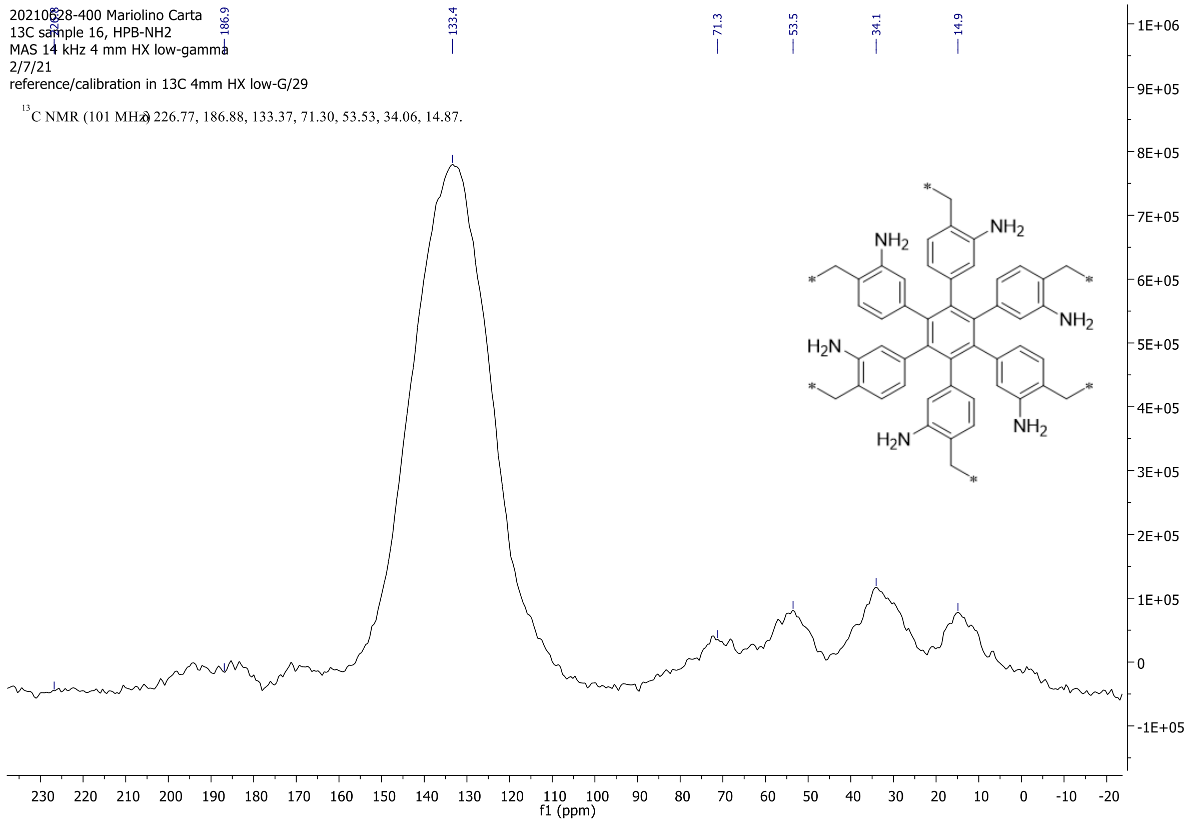

Figure SI24. ^{13}C solid state NMR for HPB-NH_2

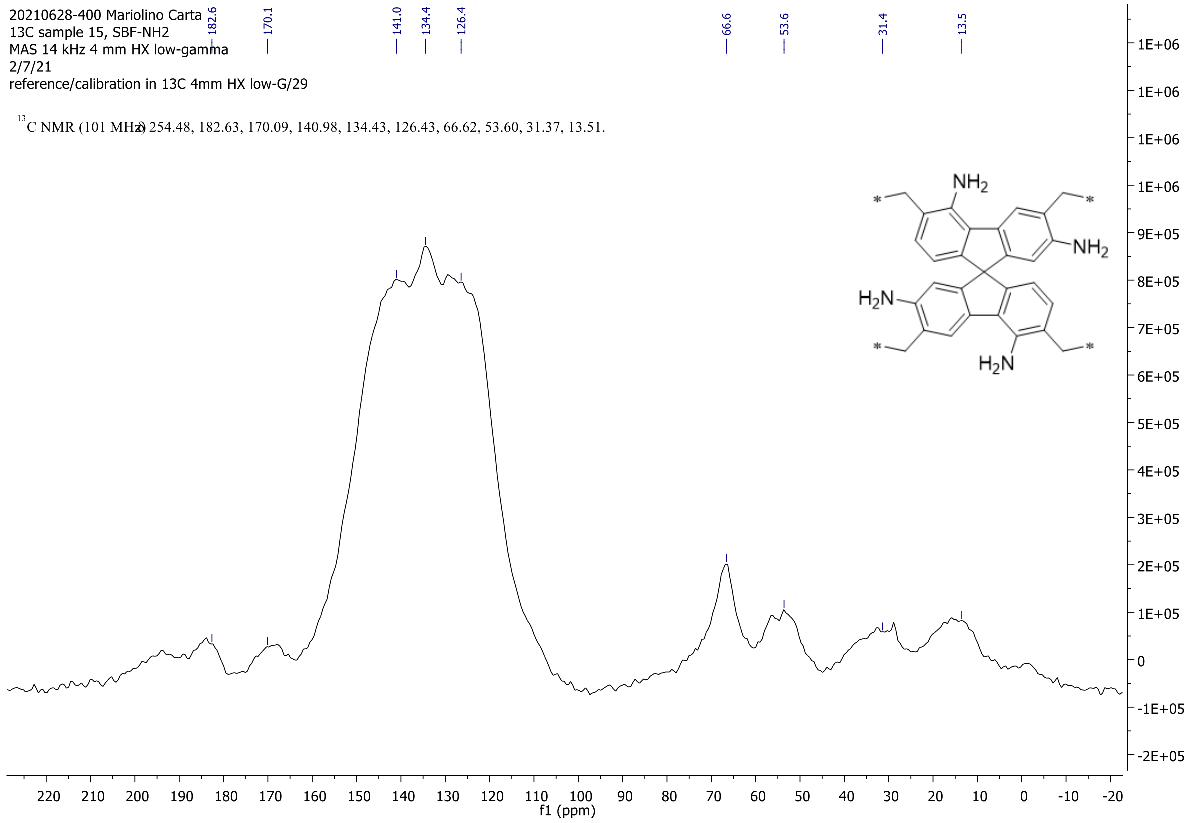

Figure SI25. ^{13}C solid state NMR for SBF-NH_2

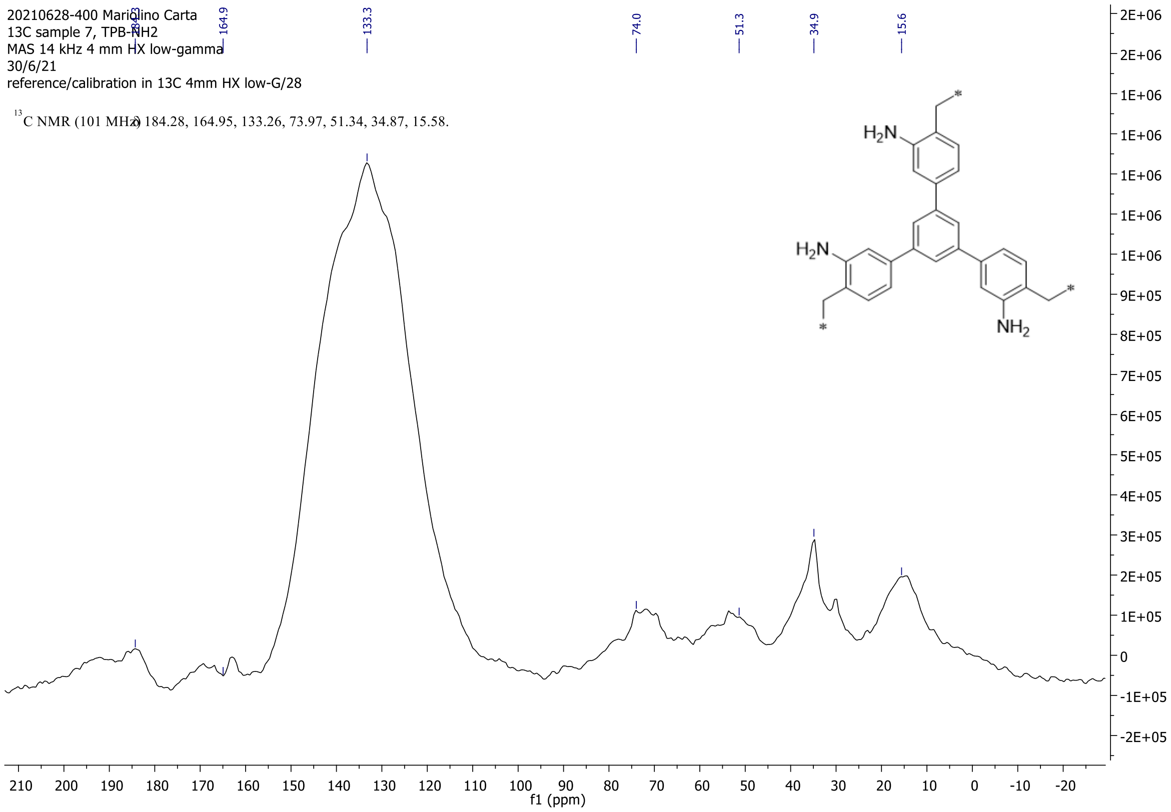

Figure SI26. ^{13}C solid state NMR for TPB-NH_2

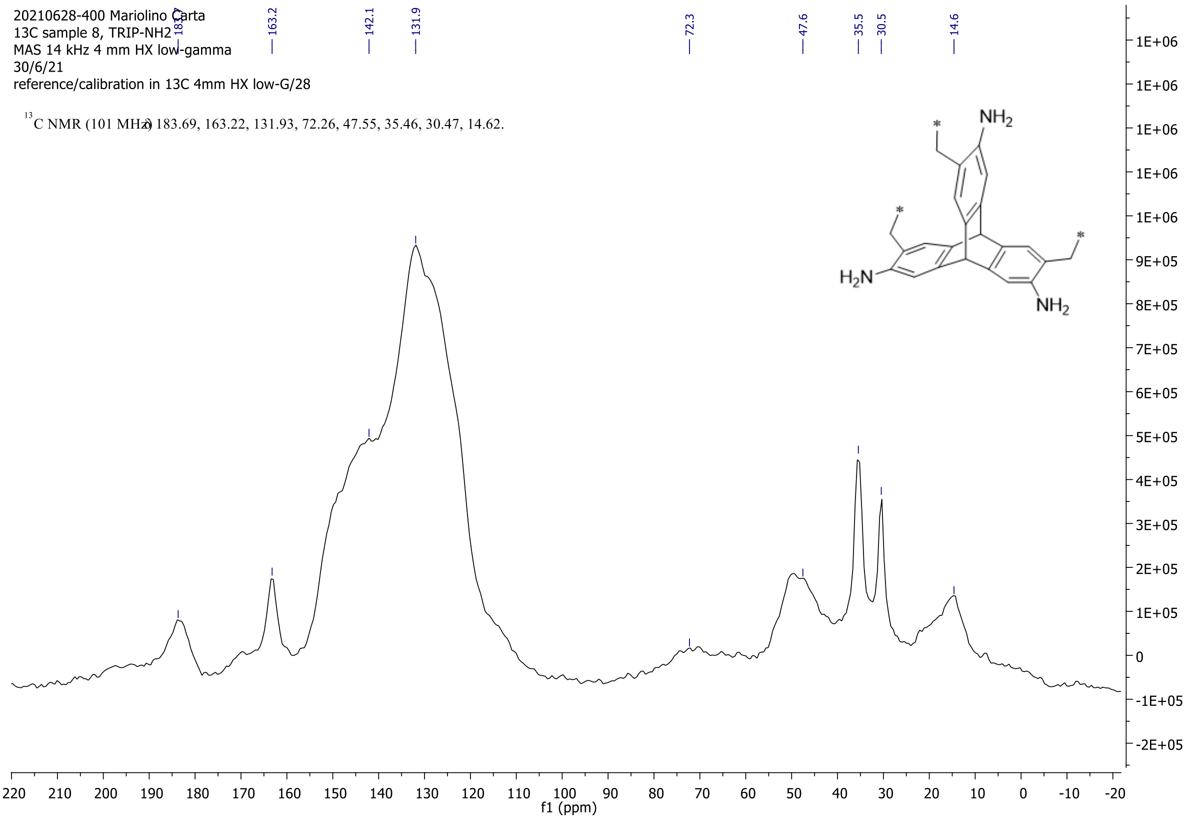

Figure SI27. ^{13}C solid state NMR for TRIP-NH_2

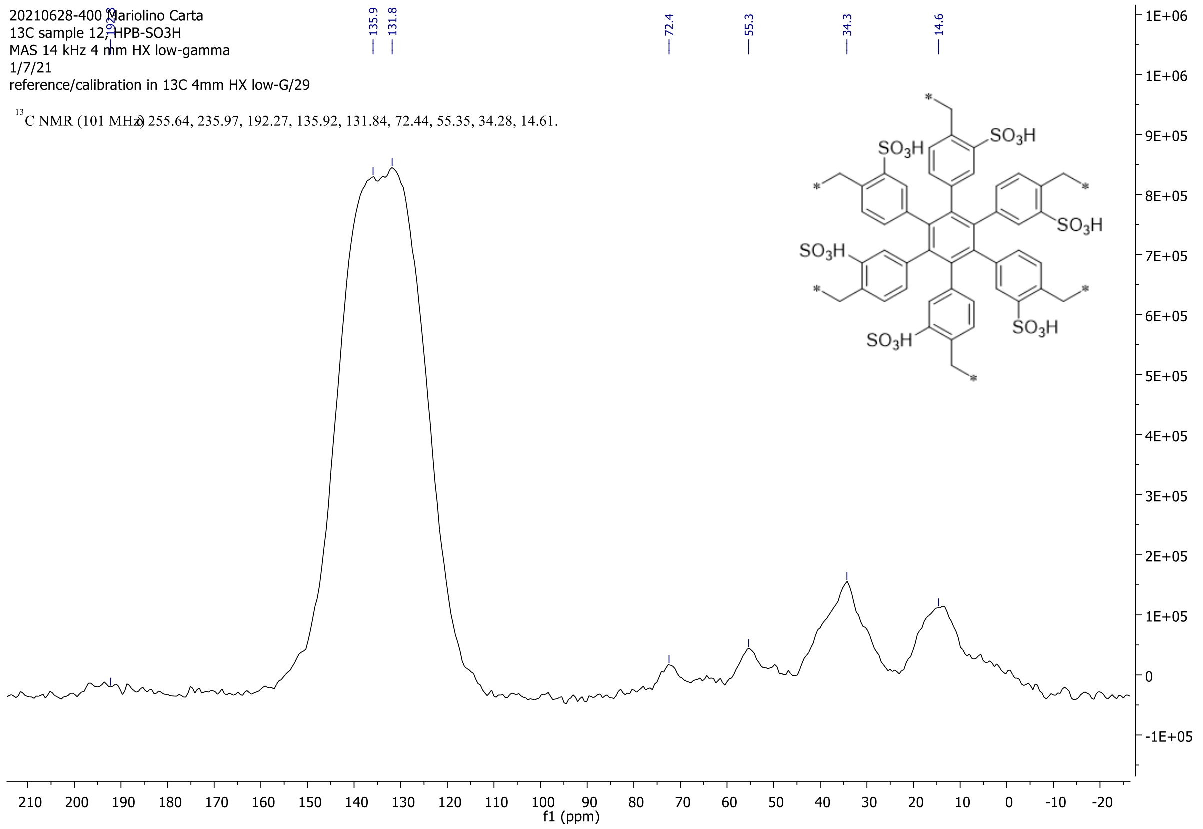

Figure SI28. ^{13}C solid state NMR for HPB-HSO$_3$

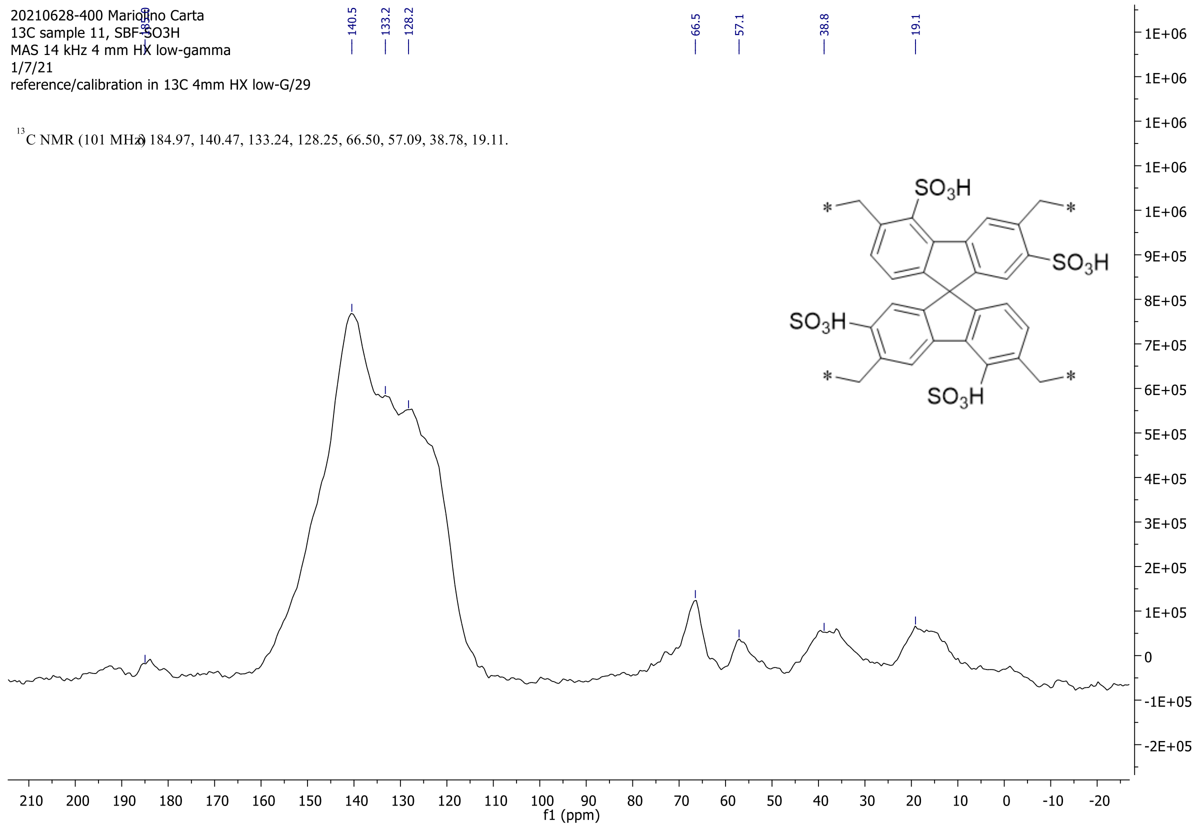

Figure SI29. ^{13}C solid state NMR for SBF-HSO$_3$

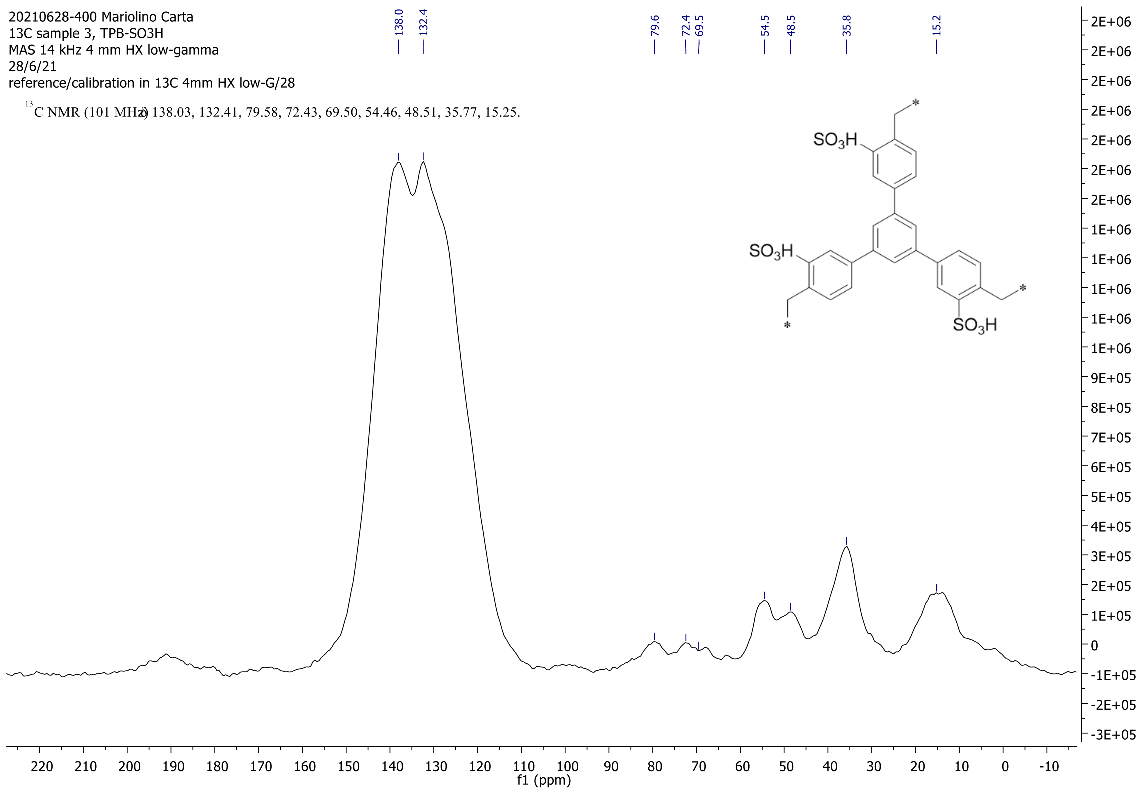

Figure SI30. ^{13}C solid state NMR for TPB-HSO_3

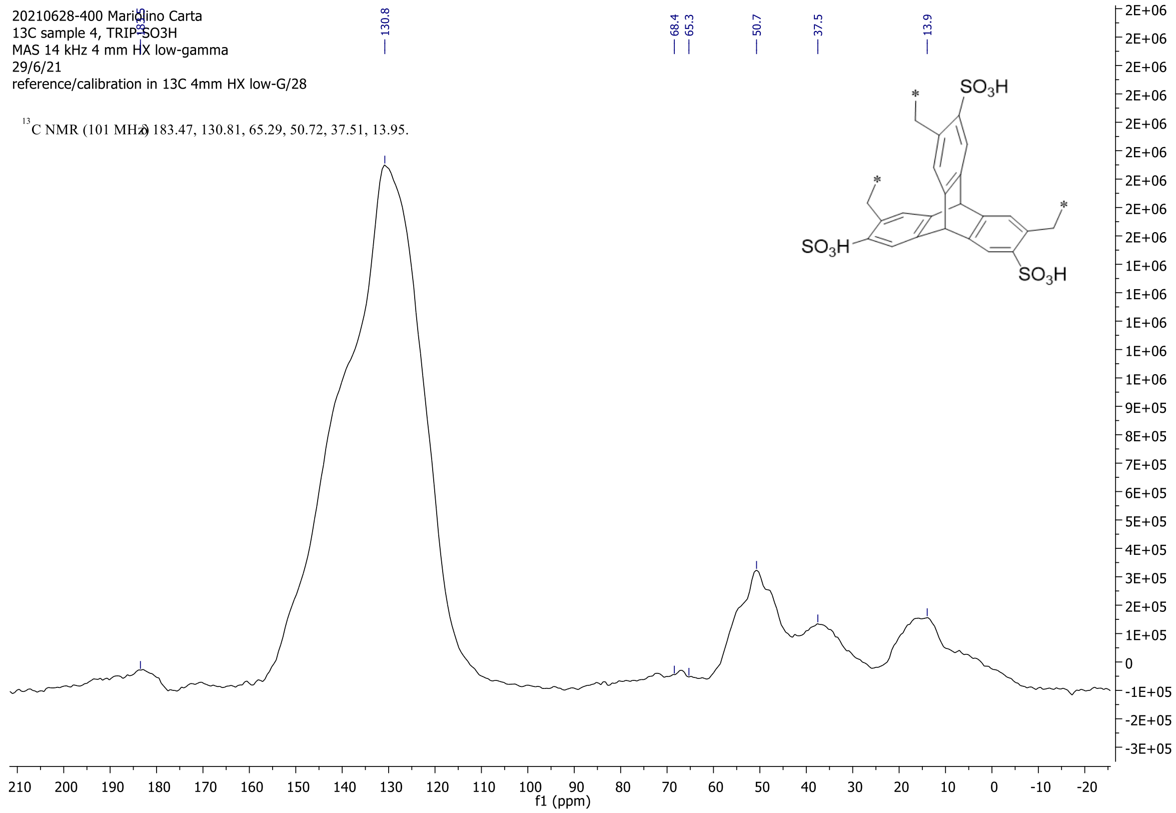

Figure SI31. ^{13}C solid state NMR for TRIP-HSO_3

10. TGA

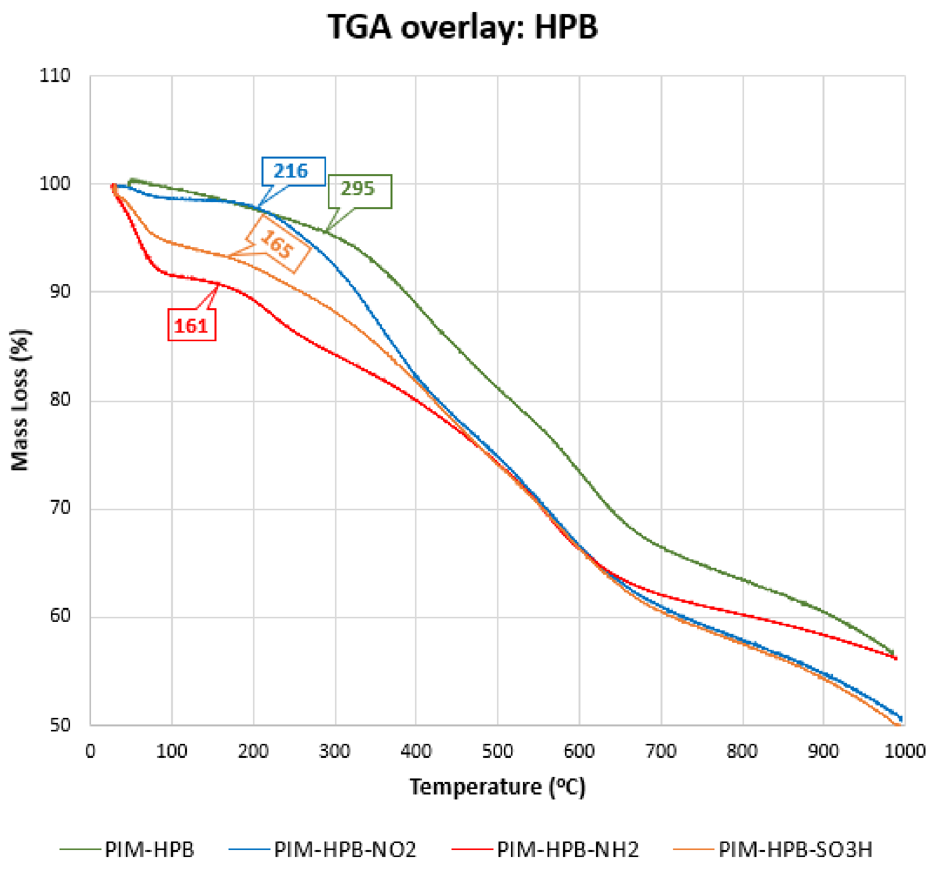

Figure SI32. TGA overlay of HPB-PIMs

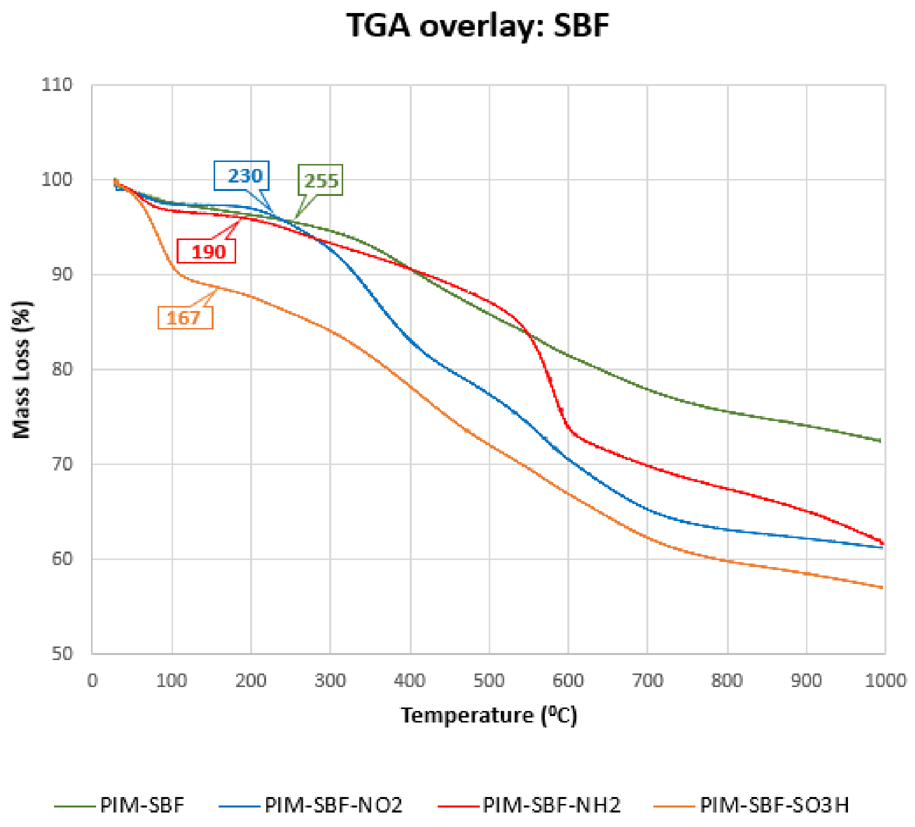

Figure SI33. TGA overlay of SBF-PIMs

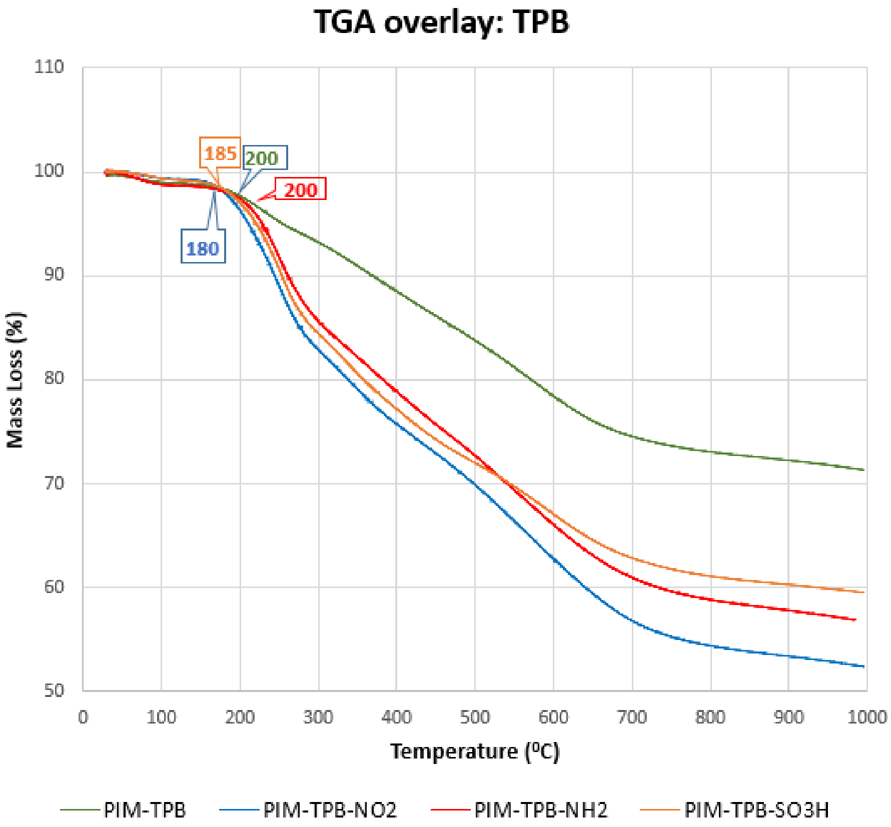

Figure SI34. TGA overlay of TPB-PIMs

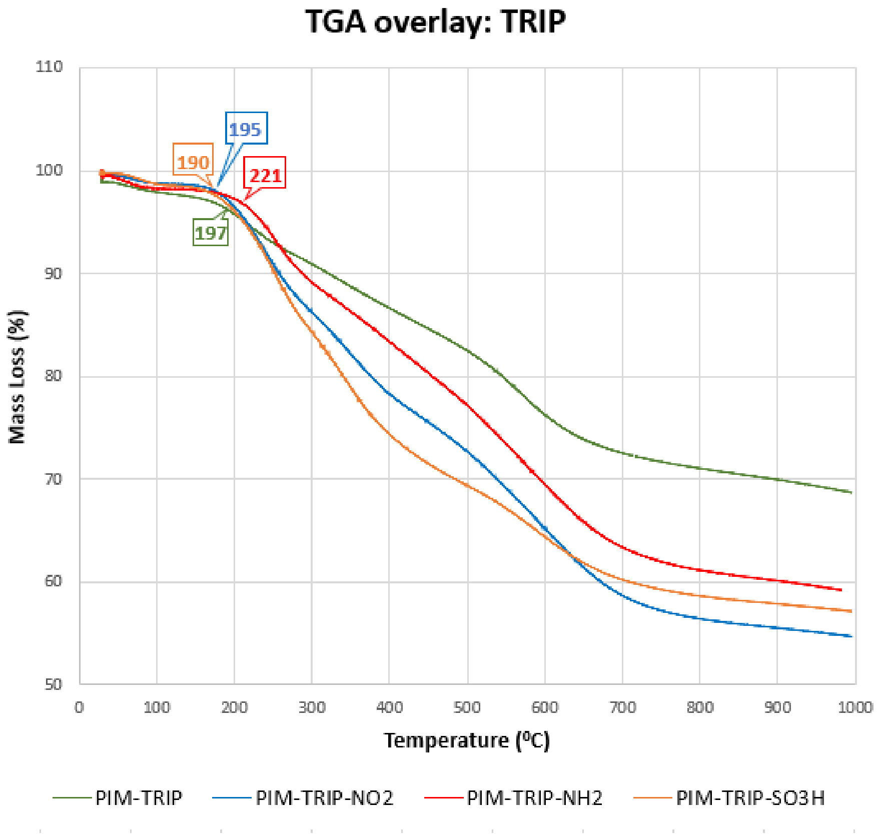

Figure SI35. TGA overlay of TRIP-PIMs

11. References

1. Myers, A. L.; Prausnitz, J. M., Thermodynamics of mixed-gas adsorption. *AIChE Journal* **1965,** *11* (1), 121-127.
2. Lee, S.; Lee, J. H.; Kim, J., User-friendly graphical user interface software for ideal adsorbed solution theory calculations. *Korean Journal of Chemical Engineering* **2018,** *35* (1), 214-221.
3. Al-Hetlani, E.; Amin, M. O.; Antonangelo, A. R.; Zhou, H.; Carta, M., Triptycene and triphenylbenzene-based polymers of intrinsic microporosity (PIMs) for the removal of pharmaceutical residues from wastewater. *Microporous and Mesoporous Materials* **2022,** *330*, 111602.
4. Msayib, K. J.; McKeown, N. B., Inexpensive polyphenylene network polymers with enhanced microporosity. *Journal of Materials Chemistry A* **2016,** *4* (26), 10110-10113.
